# *Plasmodium falciparum* Merozoite Associated Armadillo Protein (PfMAAP) Is Apically Localized in Free Merozoites and Antibodies Are Associated With Reduced Risk of Malaria

**DOI:** 10.3389/fimmu.2020.00505

**Published:** 2020-04-07

**Authors:** Yaw Aniweh, Prince B. Nyarko, Essel Charles-Chess, Felix Ansah, Faith H. A. Osier, Evelyn Quansah, Laty Gaye Thiam, Gathoni Kamuyu, Kevin Marsh, David J. Conway, Kevin K. A. Tetteh, Gordon A. Awandare

**Affiliations:** ^1^West African Centre for Cell Biology of Infectious Pathogens (WACCBIP), University of Ghana, Accra, Ghana; ^2^Department of Biochemistry, Cell and Molecular Biology, College of Basic and Applied Sciences, University of Ghana, Accra, Ghana; ^3^KEMRI-Wellcome Trust Research Programme, Centre for Geographic Medicine Research-Coast, Kilifi, Kenya; ^4^Centre for Infectious Diseases, Parasitology, Heidelberg University Hospital, Heidelberg, Germany; ^5^Department of Biochemistry, Pwani University, Kilifi, Kenya; ^6^Nuffield Department of Clinical Medicine, Centre for Tropical Medicine and Global Health, University of Oxford, Oxford, United Kingdom; ^7^Division of Medicine, Department of Respiratory Medicine, UCL, London, United Kingdom; ^8^Division of Infectious Diseases, Department of Medicine Solna, Karolinska Institutet, Stockholm, Sweden; ^9^Department of Infection Biology, London School of Tropical Medicine and Hygiene, London, United Kingdom

**Keywords:** Malaria, armadillo, invasion, merozoites, antigen, antibodies, recombinant protein

## Abstract

Understanding the functional role of proteins expressed by *Plasmodium falciparum* is an important step toward unlocking potential targets for the development of therapeutic or diagnostic interventions. The armadillo (ARM) repeat protein superfamily is associated with varied functions across the eukaryotes. Therefore, it is important to understand the role of members of this protein family in *Plasmodium* biology. The *Plasmodium falciparum* armadillo repeats only (*Pf*ARO; Pf3D7_0414900) and *P. falciparum* merozoite organizing proteins (*Pf*MOP; Pf3D7_0917000) are armadillo-repeat containing proteins previously characterized in *P. falciparum*. Here, we describe the characterization of another ARM repeat-containing protein in *P. falciparum*, which we have named the *P. falciparum* Merozoites-Associated Armadillo repeats protein (PfMAAP). Antibodies raised to three different synthetic peptides of PfMAAP show apical staining of free merozoites and those within the mature infected schizont. We also demonstrate that the antibodies raised to the PfMAAP peptides inhibited invasion of erythrocytes by merozoites from different parasite isolates. In addition, naturally acquired human antibodies to the N- and C- termini of PfMAAP are associated with a reduced risk of malaria in a prospective cohort analysis.

## Introduction

Human malaria is caused by several species of the genus *Plasmodium*, with the majority of deaths attributed to *Plasmodium falciparum*. The life cycle of the parasite is multifaceted involving both the mosquito vector and the human host, with asexual multiplication of the parasite in the blood responsible for the clinical manifestations of the disease. Asexual replication requires successful invasion of the erythrocyte by the merozoite stage. This process is complex, involving proteins released from the apical organelles or located on the merozoite surface (1, 2).

Proteins stored in organelles such as the micronemes, rhoptries, and dense granules have been extensively studied to define their roles in merozoite invasion (1, 2). Notable invasion-linked protein families include the *P. falciparum* reticulocyte binding like protein homologs (PfRH1, 2a, 2b, 4, and 5) (3–8), the erythrocyte binding antigens (PfEBAs; EBA 140, 175, 181 & EBL1) (9–14) and the rhoptry neck proteins (RONs) (15, 16). Other proteins such as AMA1 (17) have been shown to play critical roles during the process of merozoite invasion.

Our interest in the PF3D7_1035900 protein arose from a survey of the antigen-rich chromosome 10 cluster. A region that contains a number of well-characterized putative vaccine candidates including, the MSP3/6 protein family (18, 19), GLURP (20, 21), liver stage antigen 1 (22), to name but a few. In addition to its location, transcriptional data demonstrated a peak of expression at the late schizont/early merozoite stage (23, 24), a profile that hinted at a biological importance for the late schizont and merozoite stages of the parasite.

To understand the role this gene played in parasite biology our initial investigations determined that PF3D7_1035900 was a member of the armadillo protein family, a family with pleiotropic functions that warranted further investigation as a potential intervention target. The eukaryotic armadillo repeat proteins are involved in diverse roles including cell adhesion, cell motility, cytoskeletal arrangement, molecular chaperones, cell signaling/sensing, and nuclear import (25, 26). In apicomplexan parasites, the armadillo repeat containing proteins are being characterized for their role during parasite development. The importance of this protein family is highlighted by their involvement in fundamental processes essential to parasite biology, including but not restricted to gene regulation and cytokinesis. Essential processes have been linked with the previously described *P. falciparum* ARM Repeats Only (PfARO) and *P. falciparum* Merozoite Organizing Proteins (PfMOP) (22), respectively. The putative function of PfARO has been assigned through studies using the *Toxoplasma gondii* paralog, TgARO (20, 21). In this study, we describe the characterization of another member of the armadillo repeat family of proteins, encoded by gene locus PF3D7_1035900, which lies in an antigenic rich region of chromosome 10 among members of the *msp3* gene family and several other antigen genes.

The gene shows peak expression late in the developmental cycle in the schizont. Antibodies raised to synthetic peptides demonstrate staining of the apical tip in free merozoites and those within the schizont. We propose the name *Plasmodium falciparum* merozoite associated armadillo protein (PfMAAP) due to its association with fully segmented merozoites within the mature schizont and with free merozoites. Furthermore, we show that the recombinant proteins based on the N-, central repeat and C- terminal regions are recognized by antibodies in plasma of malaria exposed individuals, with antibodies to the N and C- terminal conserved domains being associated with a lower prospective risk of contracting malaria.

## Results

### PfMAAP Is an Armadillo (ARM)-Repeat Containing Protein

**Figure 1:**
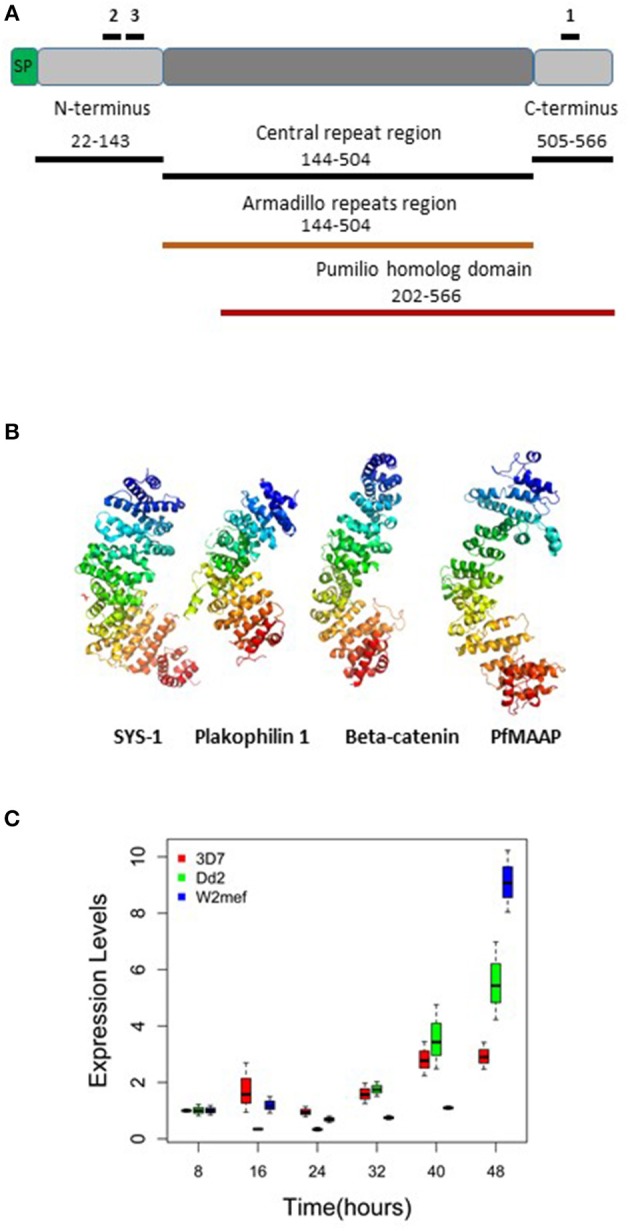
Summary of *Pf*MAAP protein characteristics, including location of peptides and recombinants and transcript expression levels. **(A)** Cartoon of the *Pf*MAAP protein highlighting the location of the *Pf*MAAP peptides, shown with black lines above the scheme and labeled 1, 2, and 3. The position of the *Escherichia coli* expressed conserved N- and C-termini and central repeat region are shown below the scheme, with black lines and corresponding amino acid positions. The Armadillo repeat region (orange, amino acid position corresponds to central repeat region) and the Pumilio homolog domain (red line, with amino acid positions shown). **(B)** The predicted structural model for *Pf*MAAP is shown as a ribbon diagram preceded by other known armadillo repeat containing proteins, Sys-1 (symmetrical sisters-1, 3C2H in Protein Data Bank), Plakophilin-1 (1XM9) and B-catenin (1JDH), highlighting the structural similarity between *Pf*MAAP and other armadillo repeat containing proteins. **(C)** Boxplots showing differential expression of *Pf*MAAP across the asexual stage of development for four laboratory isolates and one clinical isolate. Transcript fold change is plotted against time (hours post invasion), with peak expression at 40 or 48 h post merozoites invasion. Transcript experiments were conducted in triplicate and conducted on two independent occasions.

To determine the putative function of the PfMAAP protein (PF3D7_1035900) we interrogated the amino acid sequence to gain insight into the protein product. Aside from a signal peptide (amino acid 1 to 21; Figure 1A), we identified an armadillo (ARM)-repeat domain comprised of 5 repeats (aa 144-504; Figures 1A,B) and an overlapping Pumilio homolog domain (aa 202–566; Figure 1A). We analyzed the amino acid sequences for 16 isolates using the REPeats and their PERiodicities (REPPER) server (https://toolkit.tuebingen.mpg.de/#/tools/repper), which identifies short gapless repeats in both protein and nucleotide sequences (27). Both the laboratory and field isolates showed that the central repeat region always started at amino acid position 144 but varied in length from 166 (7G8 isolate) to 467 (GB4 isolate) amino acids (Figure S1 and Table S1). Amino acid sequence alignments also showed high levels of sequence conservation within the *P. falciparum* isolates (Figure S1) and between *P. falciparum* (3D7) and available sequences for non-human primate malarias at both the N- and C-terminal regions (Figure S2). The position of the repeat regions in all isolates was validated using the REPPER server (27, 28) (Figure S3 and Tables S1, S2). Further investigation of the PfMAAP protein was performed by *in silico* structural modeling using the I-Tasser structural prediction server (29–31). This was done to determine the putative structure of the proteins and to identify structural, and potentially functional, homologs of the PfMAAP protein. Using the I-Tasser structural prediction algorithm, the resolved crystal structures for three armadillo-repeat containing proteins, showing close structural homology with the PfMAAP protein were identified (Figure 1B). These include, β-catenin, a molecule shown to be involved in cadherin-based adhesion and implicated in cerebral malaria (27, 29, 30); the symmetrical sisters (SYS)-1 protein, functionally similar to β-catenin (32); and Plakophilin 1, also functionally similar to β-catenin Figure 1B). All of these which provides additional evidence of as to the potential biological significance of the PfMAAP protein.

The PfMAAP region was also identified in the available *Plasmodium* sequences from 6 *Laverania* species infections of primates (Figure S2), suggesting an ancient origin for the protein family. Using the 3D7 isolate as the reference sequence, we show that the signal peptide and an additional 100 amino acids (position 22–121) have high levels of sequence identity between the human (3D7) and *Laverania* primate species at the N- and C-terminal regions (Figure S2). However, the repeat regions varied extensively in the composition of the repeats, ranging from 190 to 402 aa in length (Figure S2 and Table S2).

### PfMAAP Is Expressed Late in Erythrocytic Stage Development

Transcript expression analysis of three *P. falciparum* laboratory-adapted strains (3D7, W2mef, Dd2) were performed across the asexual blood stage cycle at 8-h intervals. The expression levels were evaluated in triplicate and were the results of two separate experiments. The transcript expression profile demonstrated that expression of PfMAAP peaked in the later stages of parasite development at around 40–48 h post invasion (Figure 1C). This finding was supported by transcript data previously reported on Plasmodb (https://plasmodb.org/plasmo/) and in (24, 32).

### PfMAAP Is Expressed in Both Merozoites and Schizonts

**Figure 2:**
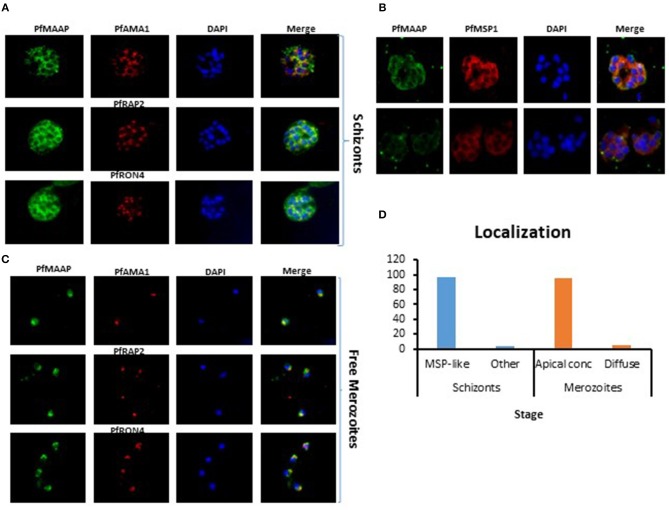
Localization of *Pf*MAAP in schizonts and merozoites. **(A)** Co-localization of anti-*Pf*MAAP peptide antibody reactivities (green) with the apical markers *Pf*AMA1 (microneme, red), *Pf*RAP2 (rhoptry bulb, red) *Pf*RON4 (rhoptry neck, red). Staining shows clear punctate apical staining for all three apical markers; **(B)** anti-*Pf*MAAP (red) co-localization with inner membrane complex (IMC) protein GAP45 and the merozoite surface markers *Pf*MSP1 (green). **(C)** The localization of anti-*Pf*MAAP antibody reactivities (green) in free merozoites relative to *Pf*AMA1, *Pf*RAP2, and *Pf*RON4. **(D)** Percentage (%) representation of the different staining patterns observed in the schizont stage (*n* = 500) as either MSP-like or any other pattern and in merozoites (*n* = 500) as either punctate apical localization or diffuse was performed using FIJI Image J. DAPI staining of the nuclei is shown in blue and the images are shown in the final column (merge). Fifty images were taken per antibody tested using an Olympus model BX41 fluorescent microscope with a x100 oil-immersion objective.

**Figure 3:**
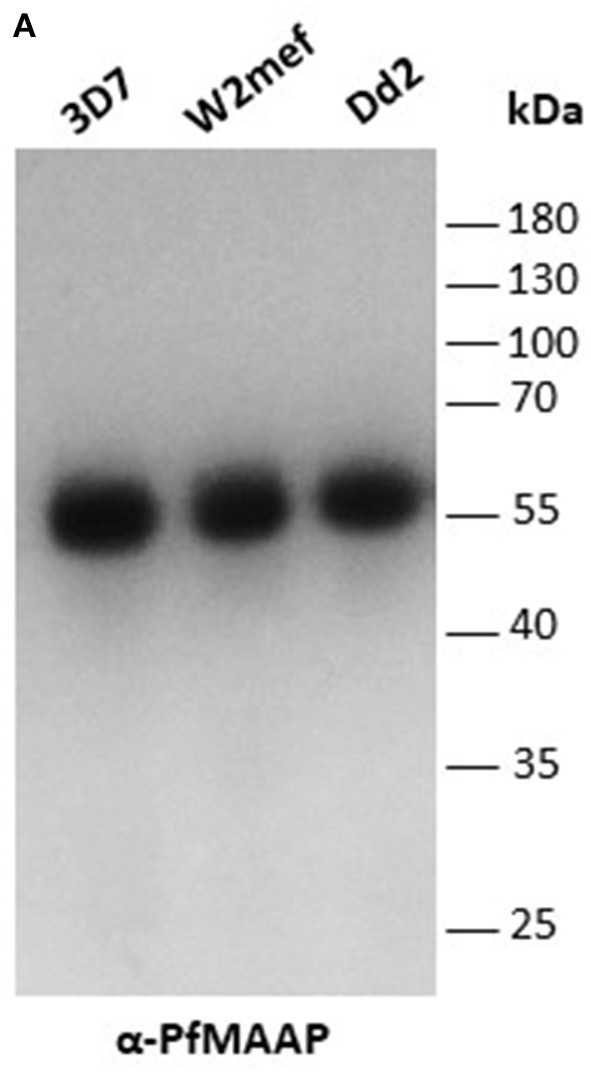
*Pf*MAAP is expressed in multiple *P. falciparum* strains. **(A)** *Pf*MAAP1 detection of the protein in Western blot from 3D7, W2mef, and Dd2 schizont extracts. Molecular weight (kDa) indicated by the side. Molecular weight (kDa) indicated by the side.

Three peptides spanning 14 amino acids, located at the C- (PfMAAP1) and N-terminal (PfMAAP2 and 3) regions (Figure 1A) were used to immunize three rabbits per group. The polyclonal sera obtained was then used to identify the location of the expressed gene product by immunofluorescence assay and thereby validate the presence of native epitopes within the peptides by recognition of the native parasite protein. Antibodies raised to all three peptides showed localization of the native protein by immunofluorescence assay (IFA) on free merozoites and those located within fixed preparations of mature schizonts (Figures 2A–C). All three antibody preparations showed similar staining patterns within mature schizonts and free merozoites (Figures 2A–C and Figure S4). As a result, all subsequent IFA experiments will simply be referred to as α-PfMAAP antibody. The staining patterns observed showed a clear merozoite surface staining pattern in developing schizonts and a predominantly apical staining in free merozoites and on those within mature rupturing schizonts (Figure 2A). The staining pattern was compared by co-localization with relative to PfAMA1 (micronemes), PfRAP2 (rhoptry bulb), and PfRON4 (rhoptry neck) (Figures 2A–C). Co-localization with α-AMA1 antibodies (a micronemal marker), showed apical staining of merozoites within the mature schizont (Figure 2A) and on free merozoites (Figure 2B), with a small proportion showing diffuse surface staining of the merozoite by the α-PfMAAP antibody (Figure 2A, top panel). Punctate apical staining was also observed with co-localization of the α-PfMAAP with the α-PfRAP2 (a marker for the rhoptry bulb) and α-PfRON4 (a rhoptry neck marker) (Figures 2A,B); although in the latter two cases, no additional peri-merozoite staining was observed. To clarify the merozoite surface-like staining observed with the α-AMA1 antibody, an additional co-localization assay was performed with an α-MSP1 antibody, as a marker for the merozoite surface. The results confirmed the merozoites surface-like staining of merozoites within the mature intact schizonts. To further evaluate the distribution of the different staining patterns, a total of 200 intact schizonts were assessed for the staining pattern; 99% (*n* = 198) showed the MSP1-like staining with 1% showing a diffuse staining pattern. The apically concentrated staining pattern observed in released merozoites was observed in 99.5% of the evaluated merozoites (*n* = 995) with the remaining 0.5% (*n* = 5) showing a diffused staining pattern (summarized in Figure 2D). In addition, we examined purified schizont extracts from three laboratory isolates (3D7, W2mef, and Dd2) by Western blot analysis to determine the relative expression of PfMAAP protein in each isolate. Screening of the blots with the α-PfMAAP antibody demonstrated that the protein was expressed in each of the three isolates tested (Figure 3), which was in keeping with transcriptomic analysis reported in (23, 24) and on Plasmodb (https://plasmodb.org/plasmo/).

### Anti-PfMAAP Antibodies Inhibit Merozoite Invasion of Erythrocytes

**Figure 4:**
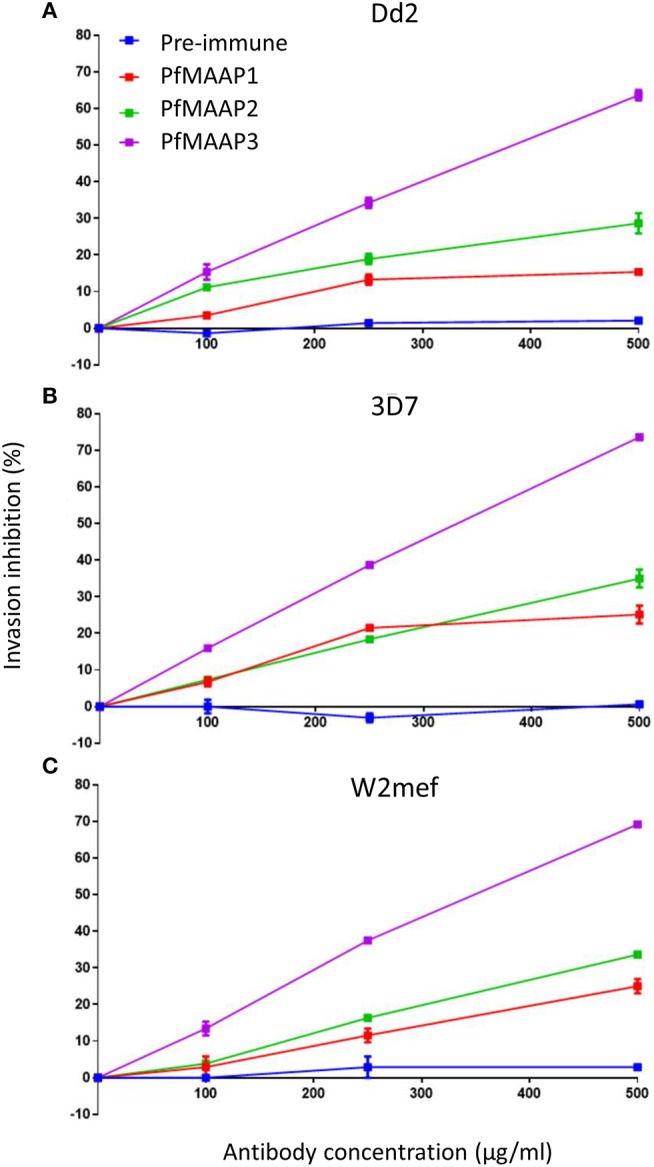
*Pf*MAAP antibodies inhibit merozoites invasion. Evaluation of the *Pf*MAAP polyclonal antibodies in a dose-dependent manner for **(A)** Dd2 **(B)** 3D7 and **(C)** W2mef parasite isolates. The antibody reactivity for each is shown, *Pf*MAAP1 (red line), 2 (green line) and 3 (purple line), with the pre-immune negative control shown in blue. Percentage (%) invasion inhibition is plotted on the y-axis with the antibody concentrations (μg/ml) plotted on the x-axis. Pre-immunization antibodies were used as the negative control. All the assays were conducted in triplicate as two separate experiments with the plots displayed as n ± sem.

To assess the potential functional importance of antibodies raised to the N- (PfMAAP2 and 3) and C-terminal (PfMAAP1) domain peptides (Figure 1A), each antibody was assessed in a growth inhibition assay (GIA). Purified schizonts from three laboratory isolates (3D7, Dd2, and W2mef) were cultured with fresh red blood cells in the presence of the antigen-specific antibodies or the equivalent amounts of purified antibodies from pre-immune sera, in a dose dependent manner (0, 100, 250, and 500 μg/ml). Both α-PfMAAP1 (C-terminal) and α-PfMAAP2 (N-terminal) inhibited parasite invasion of the Dd2 parasite strain by 15 and 25%, respectively at 0.5 mg/ml antibody concentration, whereas α-PfMAAP3 (N-terminal) inhibited parasite invasion of red blood cells by >60% at 0.5 mg/ml (Figure 4A). Similar levels of invasion inhibition were achieved for 3D7 (Figure 4B) and W2mef (Figure 4C), with the antibodies showing a dose dependent merozoites invasion inhibition.

### Antibodies Against PfMAAP Are Associated With Reduced Risk of Clinical Malaria

**Figure 5:**
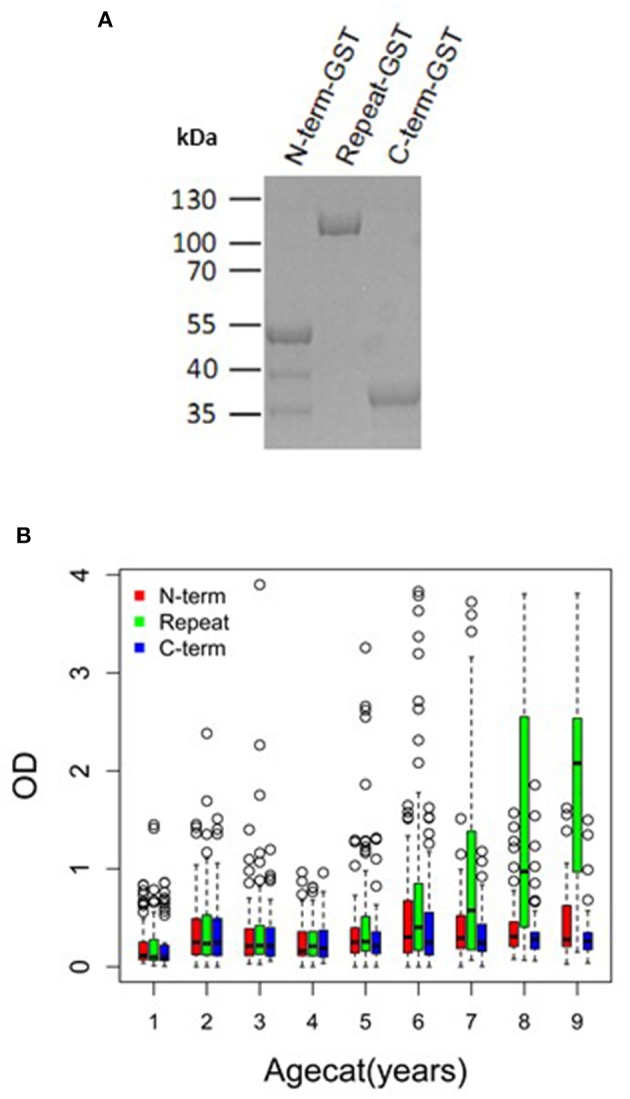
*Pf*MAAP antibodies are associated with naturally acquired immunity. **(A)** SDS PAGE showing the purified expressed and purified GST-tagged *Pf*MAAP protein regions; N-term-GST, Central Repeat-GST, and C-term-GST. Antibody responses (OD) across all ages (October 2000) to the truncated *Pf*MAAP recombinant antigens in **(B)** Chonyi (*n* = 518). Age categories are as follows: 1 = 1–2 year, *n* = 54; 2 = 3–4 year, *n* = 55; 3 = 5–6 year, *n* = 55; 4 = 7–8 year, *n* = 59; 5 = 9–10 year, *n* = 59; 6 = 11–15, *n* = 96; 7 = 16–30 *n* = 57; 8 = 31–50, *n* = 56; and 9 = 51–82, *n* = 27.

To investigate if PfMAAP might be a target of naturally acquired immunity, we expressed three recombinant proteins, based on the N- and C-terminal regions and the central polymorphic repeat region of PfMAAP (Figure 1A). Each protein was expressed in *E. coli* as soluble GST-tagged fusion proteins and resolved on SDS-PAGE (Figure 5A). ELISA was performed on plasma samples collected in Chonyi village (33) to measure IgG reactivity against each antigen fragment. The antibody responses to each antigen fragment increased with age (Figure 5B). Interestingly, the highest responses were seen to the central repeat region (Figure 5B), which corresponds to the armadillo repeat region within PfMAAP. There was a reduction in the prospective risk of clinical malaria in the subsequent 6 months after plasma collection in Chonyi associated with antibodies to both the N- and C-terminal antigens (Table 1). Although this association was not seen to the central repeat armadillo region (Table 1).

**Table 1:** Association between the presence of serum IgG to the panel of 3 antigens in children aged <11 years and parasite slide positive in October 2000 in the Chonyi village, and the occurrence of malaria over the following 6 months.

	^**a**^**Proportion of children acquiring malaria who were:**				
**Antigen**	**IgG** **positive**	**IgG** **negative**	**Univariate**	**^**b**^Multivariate**	***P***
			**IRR** **(95% CI)**	**IRR** **(95% CI)**	
^C^chonyi village (<11 years and parasite slide positive *n* = 119)					
N-term	18% (7/39)	41% (33/80)	0.44 (0.21–0.90)	0.47 (0.25–0.89)	0.021^*^
Repeat	20% (10/49)	43% (30/70)	0.48 (0.26–0.88)	0.58 (0.31–1.04)	0.068
C-term	20% (8/40)	41% (32/79)	0.49 (0.25–0.97)	0.52 (0.28–0.93)	0.028^*^

CI, Confidence Interval; IRR, Incidence Risk Ratio.

a Number of individuals developing malaria/the total number of individuals that were IgG positive or negative.

b The incidence Risk Ratio was estimated from multivariate analysis after adjusting for age and reactivity to Plasmodium falciparum schizont extract in a generalized linear models.

c Analysis focused on individuals who were parasitaemic at the time of serum sampling in October 2000.

* *P < 0.05*.

## Discussion

A critical step toward understanding the processes underlying parasite development, evasion of the immune system and the mechanisms involved in the selection and invasion of host cells, is understanding the role each protein plays in parasite biology. It is only through this that novel drug targets, diagnostics, or putative vaccine targets can be identified, and their role and relative importance be understood.

The armadillo super-repeat protein family appears to be involved in a variety of fundamental processes including cytoskeletal organization, cell-cell adhesion, organelle biogenesis, and signaling. Despite the variety of functions associated with this protein family across a number of diverse species, the key aspect they share is the presence of the tandemly arranged armadillo repeats. Investigations into the role(s) of the armadillo repeat family of proteins play in the apicomplexa is still in its infancy but there is evidence about some of the essential roles this family of proteins play during parasite development (34). With their previously derived function in eukaryotic cells (26), identifying any additional family members in *Plasmodium* spp. may illuminate different aspect of parasite biology with regards to interventions, potentially in the form of drugs, or vaccines.

To date, two armadillo repeat proteins have been described in *Plasmodium falciparum*. The *P. falciparum* armadillo-repeat only (*Pf*ARO), which bears similarity to β-catenin, an important cell-to-cell signaling molecule found in animals including humans (35). In *P. falciparum* β-catenin appears to be involved in both nuclear and rhoptry biogenesis (36). The homologous protein in *Toxoplasma gondii* (TgARO) is a multifunctional protein with similar functions to *Pf*ARO, such as rhoptry positioning and biogenesis. Importantly, *Tg*ARO can be functionally complimented by the orthologous *Pf*ARO (37–39). The *P. falciparum* merozoite organizing protein (*Pf*MOP), shown to localize to the inner membrane complex (IMC) and apical tip of the invasive merozoite stage of *P. falciparum*, appears to have a role in the biogenesis of the IMC as well as in rhoptry positioning (34, 36).

In this study we show the characterization of the *Pf*MAAP protein, initially classified as the hypothetical protein, M566. The protein was also briefly assigned to the MSP3 family, but lacked the C-terminal domain that is a defining characteristic of the family (40). We demonstrate that the protein product is expressed around 40 h post invasion, which is supported by previously published microarray proteomic and transcriptomic data (24). In keeping with the *T. gondii Tg*ARO and the *P. falciparum Pf* ARO and *Pf*MOP our results also show a clear association of *Pf*MAAP with the mature schizont and free merozoites, particularly with the apical tip.

We also demonstrate a dual localization pattern, of a punctate apical staining profile and some merozoite surface staining. The dual localization pattern observed may be associated with the myriad of functions associated with this protein family and may also be due to the early release of the *Pf*MAAP protein from the apical organelles. *Pf*MAAP has a signal peptide but lacks a transmembrane domain or a GPI-anchor and based on existing literature and sequence interrogation of the proteins there appears to be no obvious PEXEL/HT motif (41, 42) or any features indicative of a PEXEL/HT negative exported protein (PNEP) (43). Gene deletion studies of the *Pf*MAAP gene results in a reduction in growth (44) suggesting either a non-essential role in parasite development, functional redundancy or one that has a more pronounced effect elsewhere in the lifecycle.

Amino acid alignments of *P. falciparum* with available sequences for 6 non-human primate species (*Laverania* subgenus of *Plasmodium*), including *P. praefalciparum* G01 and *P. reichenowi* CDC and G01, show high levels of sequence identity at both the N- and C-termini, including the signal peptide. This level of conservation suggests a possible evolutionarily conserved function for the PfMAAP protein that has yet to be defined.

Unlike the other Armadillo repeat containing genes described in *P. falciparum, Pf* MAAP showed significant invasion inhibitory activity. Most importantly there was a statistically supported association between having antibody responses to the N- and C-terminal regions of PfMAAP and protection from malaria in Chonyi (22–53 infectious bites per year), although this association was not reflected in responses to the central repeat region. This difference in antibody responses to different parts of the same protein reflects what has been described for vaccine candidates and well-characterized markers of seroincidence in the same study sites, including MSP1 block 2, MSP1-19 and MSP2 (45, 46). The association between the conserved N- and C-terminal regions of *Pf*MAAP with *in vitro* invasion inhibition, together with the cohort study analysis strongly suggests that this protein warrants further investigation as a potential target of naturally acquired immunity. Thus, understanding the role the PfMAAP protein plays in parasite biology may yield important targets for intervention strategies.

## Materials and Methods

### Ethics Statement

Ethical approval for the use of the serum samples for use in this study had previously been obtained from the Kenyan National Ethics Committee, the University of Oxford, and the London School of Hygiene and Tropical Medicine as detailed in (47). Ethical approval for the Kenyan study on samples from human subjects was obtained from the Kenya National Research Ethics Committee, the University of Oxford, and the London School of Hygiene and Tropical Medicine. Written informed consent was obtained from a parent or guardian of each child contributing a blood sample and also from participating adults (47). Rabbit antibodies were obtained commercially by immunization under a commercial subcontract (Genscript). All animal work protocols were performed under the Association for Assessment and Accreditation of Laboratory Animal Care (AAALAC) International accreditation, following guidance written by the National Research Council of the U.S. National Academy of Sciences; and the Office for Animal Welfare (OLAW) certification, demonstrating an international commitment to responsible animal care and use.

### Parasite Cultures

*Plasmodium falciparum* laboratory isolates Dd2, W2mef, and 3D7 were cultured in complete RPMI-1640 (Sigma) (supplemented with 0.5% Albumax II (Gibco), 20 mg hypoxanthine, 2 g sodium bicarbonate (Sigma) and 0.05 mg/ml gentamicin sulfate (Sigma) using human group O^+^ erythrocytes at 4% hematocrit in a mixed gas environment (93% nitrogen, 5% CO_2_, and 2% oxygen; Air Liquide, Birmingham, United Kingdom) at 37 °C. Merozoites were purified after allowing schizonts to burst in the absence of fresh erythrocytes and pelleted at 4,000 rpm for 10 min. Parasite synchronizations were performed by treating mixed stages cultures with 5% D-Sorbitol (Sigma). Ring stages were then allowed to grow to schizonts. For tighter synchronizations, Percoll purified schizonts were allowed to invade over a 2-h period followed by Sorbitol treatment as describe above. For the time points, samples were collected every 8 h over a single cycle and stored in Trizol at −80°C freezer. RNA was extracted from each sample for subsequent gene expression analysis.

### Homology Modeling

A 3D predicted structural model of the full-length PfMAAP was obtained following submission to the I-TASSER server (https://zhanglab.ccmb.med.umich.edu/I-TASSER/) (29–31). The most robust model was further analyzed and edited using the PyMOL 2.2 software. Comparative analysis with known armadillo repeats proteins was performed using models submitted to the Protein Data Bank (PBD at https://www.rcsb.org/).

### Peptide Synthesis, Recombinant Proteins, and Polyclonal Antibody Generation

Three peptides (1-CQGEKVNKNDLNDAS, 2-FTENKEQKNEEVPMC, 3-VVNDGEEVKTEYVSC) were synthesized from the 3D7 amino acid template (Figure 1A) based on antigenicity, surface exposure and hydrophilicity scores (Genscript, US). Two of the peptides were located within the N-terminal region and the third within the C-terminal region, located in the Pumilio protein domain. Further validation of the synthesized peptide was performed by mass spectrometric analysis of the peptide sequences and HPLC analysis. Each peptide was injected at 200 μg/animal via the subcutaneous route (2 New Zealand Rabbits each) in Freunds' complete/incomplete adjuvant using a customized 48 day immunization protocol (Genscript). A pre-bleed sample was taken at day−4, with the primary immunization delivered on day 0. Booster immunisations were given on days 14 and 35, test bleeds were taken on days 21 and 42, with the protocol completed on day 48. Polyclonal antibodies were purified from the pooled sera for each of the peptides, and confirmed by ELISA titrations to a dilution limit of 1:512,000 (1.95 ng/ml). Antibodies were purified over a bed of protein A/G coupled beats and concentrated (Using Amicon 30 kDa, Merck) or diluted to the required concentration for use in the different assays they were intended.

In addition, three recombinant proteins were designed and expressed in *E. coli* as GST-tagged fusion proteins, targeting the N-, central polymorphic repeat and C-terminal regions (Figure 1A). The N- (nt 67-429) and C-terminal (nt 1513-1698) regions, both showing minimal polymorphism, and the polymorphic central repeat region (nt 430-1512) were PCR amplified from 3D7 genomic DNA. The sequence validated amplified inserts were cloned into the pGEX-2T expression vector (GE Healthcare) followed by additional sequence verification prior to transformation and expression in BL21 (DE3) *E. coli*. Expression and affinity purification were performed as described previously for other GST-fusion proteins (47). Purified proteins were assessed for purity and integrity by SDS PAGE (Figure 5A).

### Immunofluorescent Assays and Microscopy

The Protein A/G-affinity purified antibodies raised to the peptide fragments were assessed for reactivity to native parasite proteins by IFA. Synchronized late stage schizonts were smeared, air-dried and fixed with acetone (Merck) for 5 min at RT. The slides were co-incubated with rabbit anti-PfMAAP antibodies (PfMAAP 1-3) (1:500) and either anti-mouse PfAMA1 (1:1,000) (MR4), α-PfRAP2 (1:1,000), α-PfRON4 (1:400), α-PfMSP1 or α-PfGAP45, respectively, for 1 h in 3% BSA/PBS buffer, followed by three washes in 1X PBS at 5 min/wash. The slides were then incubated with Alexa Flour 594 goat anti-mouse IgG (H+L) (red) and Alexa Flour 488 goat anti-rabbit IgG (H+L) (red) secondary antibodies (1:1,000 respectively) (Molecular Probes) for 1 h. Slides were washed in 1X PBS three times for 5 min each and air-dried. Mounting medium containing DAPI (Vector laboratories) was added to each slide and sealed with a coverslip for microscopy. The cells were imaged on an Olympus System Microscope Model BX41 with a Hamamatsu ORCA-spark Digital CMOS camera C11440-36U. In total 50 images were taken for each antibody used. All image background subtraction, brightness and contrast adjustment as well as all analysis were conducted using Fiji ImageJ software.

### Western Blot Analysis of Parasite Extracts and Culture Supernatants

Ring stage cultures were synchronized with 5% D-sorbitol (Sigma) and cultured to mature stage schizonts (48, 49), followed by purification over a Percoll gradient (50). The purified schizonts were washed twice with 1xPBS and resuspended in 1xPBS. Aliquots were lysed in SDS PAGE sample buffer, resolved using a 12% SDS-PAGE before transfer onto nitrocellulose membranes (0.2 μm, Bio-Rad). Proteins were detected using the PfMAAP1 & 2 polyclonal antibodies (pAbs) @ 1:1,000 dilution. Following primary incubation, the blots were washed and incubated with anti-rabbit HRP-conjugated secondary antibodies @ 1:3,000 dilution for 1 h followed by two washes with 1X PBS. The membrane was developed using enhanced chemiluminescence (GE healthcare) and developed using the KODAK image analysis system.

### Merozoites Invasion Inhibition Assay

Synchronized late-stage schizonts were purified, and assays were plated as previously described (50). Briefly, target cells (erythrocyte acceptor cells) were stained with 5-(and-6)-carboxyfluorescein diacetate succinimidyl ester at 20 μM (5(6)CFDA-SE; Invitrogen), a cytoplasmic fluorescent stain, to help differentiate erythrocytes invaded in the assay from those in the parasite inoculum. Late stage parasites at 2% parasitemia were mixed with the 5(6)CFDA-SE-labeled erythrocyte acceptor cells in a 1:1 ratio at 2% hematocrit in 100 μl assays in 96-well titer plates. Increasing concentrations of purified anti-peptide antibody for PfMAAP peptides 1- 3 (0–500 μg/ml) were added to corresponding wells. Control wells were incubated with pre-immune sera. Assays plates were incubated overnight at 37°C in a mixed gas environment to allow one cycle of invasion. Cells were stained with Hoechst 33342 (Sigma Aldrich) and washed 3 times with complete RPMI. Invaded target cells were counted with a BD LSR Fortessa X20 flow cytometer. The experiment was run twice independently with each condition conducted in triplicate. A total of 50,000 RBCs were counted and % invasion into the 5(6)CFDA-SE-labeled target cells were recorded. The percentage of successfully invaded RBCs in the presence of anti-PfMAAP antibodies or the pre-immune controls were compared with the level of invasion in control wells without antibody added.

### Expression Levels of PfMAAP

The expression of the PfMAAP were assayed in three laboratory isolates (3D7, Dd2 and W2mef), following 6, 8-h sample collection time points. The expression analysis assay protocol was as described by Baker et al. (51). Briefly, RNA was purified from tightly synchronized parasites as described previously, using the AllPrep DNA/RNA Mini kit protocol by Qiagen (Qiagen, Germany) and treated with DNase to remove all traces of DNA in the sample. The cDNA synthesis was then carried out with a control reaction (without a reverse transcriptase) using the Superscript III first-strand protocol following manufacturer's instructions (ThermoFisher Scientific, USA). The cDNA synthesis reaction conditions were as follows: 25°C for 10 min, 50°C for 50 min and 85°C for 5 min. Following the cDNA synthesis, 1 μl of RNase H was added to each reaction, mixed and incubated at 37°C for 20 min followed by 95°C for 10 min to remove unconverted RNA molecules. The quantitation of the transcript level of PfMAAP gene was evaluated relative to the 60S ribosomal protein L18-2 (PF3D7_1341300) (51); a housekeeping gene used as a control. Each experiment was conducted in triplicate and conducted at two independent times. The expression levels were calculated from the Ct values using the 2^−ΔΔCt^ formula.

### Analysis of Naturally Acquired Antibodies

The plasma samples analyzed in the study were previously collected as part of a community cohort study undertaken in Chonyi, Kenya. A village in Kilifi district near the eastern coast of Kenya. The inhabitants were naturally exposed to biannual peaks of transmission in November to December and May to July, with moderate rates of transmission at the time of sampling (22–53 infectious bites/person/year; October 2000) (33). Active and passive case detection was used to determine the occurrence of episodes of clinical malaria in the following 6-month period within the communities. Indirect ELISAs were performed with each of the three antigens using protocols as previously described (47). Briefly, antigens coated at 50 ng/well were in duplicate with sera diluted to 1/500. Due to the fact that the proteins used in the assay were generated as GST-tagged fusion proteins, purified GST was included in the assay and results subtracted to correct for any background reactivity to the tag. Samples were scored as positive if the ELISA optical density (OD) values were higher than the mean plus 3 standard deviations of the values from 20 malaria-naive control sera tested in parallel (the same panel of negative-control sera was used in all assays). The risk of clinical malaria and association with antibody status was analyzed for subjects who were asymptomatic and <11 years of age at the time of sampling (Chonyi N=119/518), as done previously in analyses of antibodies to other antigens (47).

### Data Analysis

Boxplots were generated in R (R studio, version 3.5.2). Generalized linear models (GLM) were used to determine the risk ratio (RR) associated with the presence or absence of detectable serum antibodies (IgG above the cut-off OD value) and the occurrence of subsequent clinical malaria episodes. Age and antibody reactivity to parasite schizont extract were used in multivariate analyses to correct for the confounding effects of exposure on antibody responses. Statistical analyses were performed using Stata/IC (StataCorp LP, USA).

## Data Availability Statement

All datasets generated for this study are included in the article/Supplementary Material.

## Author Contributions

YA, GA, and KT: conceptualization. YA, GA, KT, DC, and FO: designed experiments. YA, PN, EC-C, FA, EQ, GK, LT, and KT: performed experiments. YA, KT, PN, and FA: analyzed the data. KT, GA, DC, and KM: supervised the study. YA, KT, GA, and DC: wrote the manuscript. All authors read and agreed to the final manuscript.

### Conflict of Interest

The authors declare that the research was conducted in the absence of any commercial or financial relationships that could be construed as a potential conflict of interest. The reviewer LK declared a shared affiliation, with no collaboration, with one of the authors, KM, to the handling editor at the time of the review.

## Supplementary Material

Figure S1 Amino acid sequence alignment of full-length sequences from 16 Plasmodium falciparum isolates for PfMAAP (PF3D7_1035900, PfSD01_100040400, PfML01_100039900, PfKE01_100041100, PfIT_100039800, PfGB4_100040700, PfGN01_100041300, PfTG01_100041000, PfDd2_100041100, PfSN01_100041200, PfKH02_100041200, Pf7G8_100040200, PfHB3_100040200, PfGA01_100041100, PfKH01_100040300, PfCD01_100041000). Alignments were generated using Clustal Omega (Sievers et al., 2011). All sequences were obtained from Plasmodb (https://plasmodb.org/plasmo/).

Figure S2 Amino acid alignment of full-length sequences from non-malaria isolates for PfMAAP (PF3D7_1035900) aligned with the 3D7 isolate as a reference. Alignments were generated using Clustal Omega (Sievers et al., 2011). *Plasmodium falciparum*, PF3D7_1035900; *Plasmodium rechenowi*, PRG01_1034400 and PRCDC_1035200; *Plasmodium billcolllinsi*, PBILCG01_1034800; *Plasmodium gaboni*, PGSY75_0012400; *Plasmodium praefalciparum*, PPRFG01_1036900; and *Plasmodium adleri*, PADL01_1034600. All sequences were obtained from Plasmodb (https://plasmodb.org/plasmo/).

Figure S3 N- and C-terminal semi-conserved and armadillo repeat sequences for PfMAAP in **(A)** sixteen laboratory adapted isolates (PF3D7_1035900, PfSD01_100040400, PfML01_100039900, PfKE01_100041100, PfIT_100039800, PfGB4_100040700, PfGN01_100041300, PfTG01_100041000, PfDd2_100041100, PfSN01_100041200, PfKH02_100041200, Pf7G8_100040200, PfHB3_100040200, PfGA01_100041100, PfKH01_100040300, PfCD01_100041000) and **(B)** in five *Laverania* subgenus of *Plasmodium* (*P.rechenowi*, PRG01_1034400 and PRCDC_1035200; *P.billcolllinsi*, PBILCG01_1034800; *P. gaboni*, PGSY75_0012400; *P.praefalciparum*, PPRFG01_1036900; and *P. adleri*, PADL01_1034600. All sequences were obtained from Plasmodb (https://plasmodb.org/plasmo/).

Figure S4 Co-localization of α-*Pf*MAAP1 and 2 with α-AMA1. **(A)** *Pf*MAAP1 (green) localization pattern relative to *Pf*AMA1 (red) with DAPI (blue) staining for nuclei and the merging of all channels (Merge). **(B)** *Pf*MAAP2 (green) co-localization with *Pf*AMA1 (red) with DAPI (blue) staining the nuclei and the merge of all channels (Merge). Fifty images were taken per antibody tested using an Olympus model BX41 fluorescent microscope with a x100 oil-immersion objective.
