# Supplementary material for: Plasmodium falciparum Merozoite Associated Armadillo Protein (PfMAAP) Is Apically Localized in Free Merozoites and Antibodies Are Associated With Reduced Risk of Malaria
Source: Front Immunol. 2020 Apr 7;11:505. doi: 10.3389/fimmu.2020.00505 (PMC7155890; doi:10.3389/fimmu.2020.00505)
Supplement: Figure S1 — Amino acid sequence alignment of full-length sequences from 16 Plasmodium falciparum isolates for PfMAAP (PF3D7_1035900, PfSD01_100040400, PfML01_100039900, PfKE01_100041100, PfIT_100039800, PfGB4_100040700, PfGN01_100041300, PfTG01_100041000, PfDd2_100041100, PfSN01_100041200, PfKH02_100041200, Pf7G8_100040200, PfHB3_100040200, PfGA01_100041100, PfKH01_100040300, PfCD01_100041000). Alignments were generated using Clustal Omega (Sievers et al., 2011). All sequences were obtained from Plasmodb (https://plasmodb.org/plasmo/). [file Image_1.pdf]

Figure S1. Figure S1 | Amino acid sequence alignment of full-length sequences from 16 *Plasmodium falciparum* isolates for PfMAAP (PF3D7_1035900, PfSD01_100040400, PfML01_100039900, PfKE01_100041100, PfIT_100039800, PfGB4_100040700, PfGN01_100041300, PfTG01_100041000, PfDd2_100041100, PfSN01_100041200, PfKH02_100041200, Pf7G8_100040200, PfHB3_100040200, PfGA01_100041100, PfKH01_100040300, PfCD01_100041000). Alignments were generated using Clustal Omega (Sievers et al., 2011). All sequences were obtained from Plasmodb (https://plasmodb.org/plasmo/).

```
PF3D7_1035900        MLNIFNIIFLLFLINIYICEANGTLSENIESAEEIDALKTNLRNGYLNNTYFNEENNNLN 60
PfSD01_100040400     MLNIFNIIFLLFLINIYICEANGTLSENIESAEEIDALKTNLRNGYLNNTYFNEENNNLN 60
PfML01_100039900     MLNIFNIIFLLFLINIYICEANGTLSENIESAEEIDALKTNLRNGYLNNTYFNEENNNLN 60
PfKE01_100041100     MLNIFNIIFLLFLINIYICEANGTLSENIESAEEIDALKTNLRNGYLNNTYFNEENNNLN 60
PfIT_100039800       MLNIFNIIFLLFLINIYICEANGTLSENIESAEEIDALKTNLRNGYLNNTYFNEENNNLN 60
PfGB4_100040700      MLNIFNIIFLLFLINIYICEANGTLSENIESAEEIDALKTNLRNGYLNNTYFNEENNNLN 60
PfGA01_100041100     MLNIFNIIFLLFLINIYICEANGTLSENIESAEEIDALKTNLRNGYLNNTYFNEENNNLN 60
PfGN01_100041300     MLNIFNIIFLLFLINIYICEANGTLSENIESAEEIDALKTNLRNGYLNNTYFNEENNNLN 60
PfTG01_100041000     MLNIFNIIFLLFLINIYICEANGTLSENIESAEEIDALKTNLRNGYLNNTYFNEENNNLN 60
PfDd2_100041100      MLNIFNIIFLLFLINIYICEANGTLSENIESAEEIDALKTNLRNGYLNNTYFNEENNNLN 60
PfSN01_100041200     MLNIFNIIFLLFLINIYICEANGTLSENIESAEEIDALKTNLRNGYLNNTYFNEENNNLN 60
PfCD01_100041000     MLNIFNIIFLLFLINIYICEANGTLSENIESAEEIDALKTNLRNGYLNNTYFNEENNNLN 60
PfKH02_100041200     MLNIFNIIFLLFLINIYICEANGTLSENIESAEEIDALKTNLRNGYLNNTYFNEENNNLN 60
Pf7G8_100040200      MLNIFNIIFLLFLINIYICEANGTLSENIESAEEIDALKTNLRNGYLNNTYFNEENNNLN 60
PfHB3_100040200      MLNIFNIIFLLFLINIYICEANGTLSENIESAEEIDALKTNLRNGYLNNTYFNEENNNLN 60
PfKH01_100040300     MLNIFNIIFLLFLINIYICEANGTLSENIESAEEIDALKTNLRNGYLNNTYFNEENNNLN 60
                     ************************************************************

PF3D7_1035900        IENEINNTNYNEVTEETKEELYDINENIFPDYFFLDIFTENKEQKNEEVPMKIEVVNDGE 120
PfSD01_100040400     IENEINNTNYNEVTEETKEELYDINENIFPDYFFLDIFTENKEQKNEEVPMKIEVVNDGE 120
PfML01_100039900     IENEINNTNYNEVTEETKEELYDINENIFPDYFFLDIFTENKEQKNEEVPMKIEVVNDGE 120
PfKE01_100041100     IENEINNTNYNEVTEETKEELYDINENIFPDYFFLDIFTENKEQKNEEVPMKIEVVNDGE 120
PfIT_100039800       IENEINNTNYNEVTEETKEELYDINENIFPDYFFLDIFTENKEQKNEEVPMKIEVVNDGE 120
PfGB4_100040700      IENEINNTNYNEVTEETKEELYDINENIFPDYFFLDIFTENKEQKNEEVPMKIEVVNDGE 120
PfGA01_100041100     IENEINNTNYNEVTEETKEELYDINENIFPDYFFLDIFTENKEQKNEEVPMKIEVVNDGE 120
PfGN01_100041300     IENEINNTNYNEVTEETKEELYDINENIFPDYFFLDIFTENKEQKNEEVPMKIEVVNDGE 120
PfTG01_100041000     IENEINNTNYNEVTEETKEELYDINENIFPDYFFLDIFTENKEQKNEEVPMKIEVVNDGE 120
PfDd2_100041100      IENEINNTNYNEVTEETKEELYDINENIFPDYFFLDIFTENKEQKNEEVPMKIEVVNDGE 120
PfSN01_100041200     IENEINNTNYNEVTEETKEELYDINENIFPDYFFLDIFTENKEQKNEEVPMKIEVVNDGE 120
PfCD01_100041000     IENEINNTNYNEVTEETKEELYDINENIFPDYFFLDIFTENKEQKNEEVPMKIEVVNDGE 120
PfKH02_100041200     IENEINNTNYNEVTEETKEELYDINENIFPDYFFLDIFTENKEQKNEEVPMKIEVVNDGE 120
Pf7G8_100040200      IENEINNTNYNEVTEETKEELYDINENIFPDYFFLDIFTENKEQKNEEVPMKIEVVNDGE 120
PfHB3_100040200      IENEINNTNYNEVTEETKEELYDINENIFPDYFFLDIFTENKEQKNEEVPMKIEVVNDGE 120
PfKH01_100040300     IENEINNTNYNEVTEETKEELYDINENIFPDYFFLDIFTENKEQKNEEVPMKIEVVNDGE 120
                     ************************************************************
```

Figure S1. Continued.

```
PF3D7_1035900           EVKTEYVSEKNEEVENKSETEIGEELTEKVDEKVPEEVAEELVEKVDEEVAEELVEKVDE 180
PfSD01_100040400        EVKTEYVSEKNEEVENKSETEIGEELTEKVDEKVPEEVAEELVEKVDEEVAEELVEKVDE 180
PfML01_100039900        EVKTEYVSEKNEEVENKSETEIGEELTEKVDEKVPEEVAEELVEKVDEEVAEELVEKVDE 180
PfKE01_100041100        EVKTEYVSEKNEEVENKSETEIGEELTEKVDEKVPEEVAEELVEKVDEEVAEELVEKVDE 180
PfIT_100039800          EVKTEYVSEKNEEVENKSETEIGEELTEKVDEKVPEEVAEELVEKVDEEVAEELVEKVDE 180
PfGB4_100040700         EVKTEYVSEKNEEVENKSETEIGEELTEKVDEKVPEEVAEELVEKVDEEVAEELVEKVDE 180
PfGA01_100041100        EVKTEYVSEKNEEVENKSETEIGEELTEKVDEKVPEEVAEELVEKVDEEVAEELVEKVDE 180
PfGN01_100041300        EVKTEYVSEKNEEVENKSETEIGEELTEKVDEKVPEEVAEELVEKVDEEVAEELVEKVDE 180
PfTG01_100041000        EVKTEYVSEKNEEVENKSETEIGEELTEKVDEKVPEEVAEELVEKVDEEVAEELVEKVDE 180
PfDd2_100041100         EVKTEYVSEKNEEVENKSETEIGEELTEKVDEKVPEEVAEELVEKVDEEVAEELVEKVDE 180
PfSN01_100041200        EVKTEYVSEKNEEVENKSETEIGEELTEKVDEKVPEEVAEELVEKVDEEVAEELVEKVDE 180
PfCD01_100041000        EVKTEYVSEKNEEVENKSETEIGEELTEKVDEKVPEEVAEELVEKVDEEVAEELVEKVDE 180
PfKH02_100041200        EVKTEYVSEKNEEVENKSETEIGEELTEKVDEKVPEEVAEELVEKVDEEVAEELVEKVDE 180
Pf7G8_100040200         EVKTEYVSEKNEEVENKSETEIGEELTEKVDEKVPEEVAEELVEKVDEEVAEELVEKVDE 180
PfHB3_100040200         EVKTEYVSEKNEEVENKSETEIGEELTEKVDEKVPEEVAEELVEKVDEEVAEELVEKVDE 180
PfKH01_100040300        EVKTEYVSEKNEEVENKSETEIGEELTEKVDEKVPEEVAEELVEKVDEEVAEELVEKVDE 180
                        ************************************************************

PF3D7_1035900           KVAEEVDQKVDEEVTEELIEKVDEEVTEELIEKVDEEVAEELIEKVDEEVAEELIEKVAD 240
PfSD01_100040400        KVAEEVDQKVDEEVTEELIEKVDEEVTEELIEKVDEEVAEELIEKVDEEVAEELIEKVAD 240
PfML01_100039900        KVAEEVDQKVDEEVTEELIEKVDEEVTEELIEKVDEEVAEELIEKVDEEVAEELIEKVAD 240
PfKE01_100041100        KVAEEVDQKVDEEVTEELIEKVDEEVTEELIEKVDEEVAEELIEKVADELVEKVAEELVE 240
PfIT_100039800          K----VDQKVDEEVTEELIEKVDEEVTEELIEKVDEEVAEELIEKVDEEVAEELIEKVAD 236
PfGB4_100040700         KVAEEVDQKVDEEVTEELIEKVDEEVTEELIEKVDEEVAEELIEKVDEEVAEELIEKVAD 240
PfGA01_100041100        KVAEEVDQKVDEEVTEELIEKVDEEVTEELIEKVDEEVAEELIEKVDEEVAEELIEKVAD 240
PfGN01_100041300        KVAEEVDQKVDEEVTEELIEKVDEEVTEELIEKVDEEVAEELIEKVDEEVAEELIEKVAD 240
PfTG01_100041000        KVAEEVDQKVDEEVTEELIEKVDEEVTEELIEKVDEEVAEELIEKVDEEVAEELIEKVAD 240
PfDd2_100041100         KVAEEVDQKVDEEVTEELIEKVDEEVTEELIEKVDEEVAEELIEKVDEEVAEELIEKVAD 240
PfSN01_100041200        KVAEEVDQKVDEEVTEELIEKVDEEVTEELIEKVDEEVAEELIEKVDEEVAEELIEKVAD 240
PfCD01_100041000        KVAEEVDQKVDEEVTEELIEKVDEEVTEELIEKVDEEVAEELIEKVDEEVAEELIEKVAD 240
PfKH02_100041200        KVAEEVDQKVDEEVTEELIEKVDEEVTEELIEKVDEEVAEELIEKVDEEVAEELIEKVAD 240
Pf7G8_100040200         KV----DQKVDEEVTEELI------------EKVDEEVTEELIEKVDEEVAEELIEKVAD 224
PfHB3_100040200         KVAEEVDQKVDEEVTEELIEKVDEEVTEELIEKVDEEVAEELIEKVDEEVAEELIEKVAD 240
PfKH01_100040300        KVAEEVDQKVDEEVTEELIEKVDEEVTEELIEKVDEEVAEELIEKVDEEVAEELIEKVAD 240
                        *     *************            *******:******* :*:.*:: *::.:
```

Figure S1. Continued.

```
PF3D7_1035900          ELIEKVDEEVAEELIEKVADELVEKVAEELVEKVDEEVAEELVEKVDEKVAEEVD----- 295
PfSD01_100040400       ELVEKVAEELVEKVDEQVAEELVE--------KVDEQVAEELVEKVDEQVVEEVADELVE 292
PfML01_100039900       ELVEKVAEELVEKVDEQVAEELVE--------KVDEQVAEELVEKVDEQVVEEVADELVE 292
PfKE01_100041100       KVDEQVAEELVEKVDEQVAEELVE--------KVDEQVAEELVEKVDEQVVEEVADELVE 292
PfIT_100039800         ELVEKVAEELVEKVDEQVAEELVE--------KVDEQV---------------------- 266
PfGB4_100040700        ELVEKVAEELVEKVDEQVAEELVE--------KVDEQVAEELVEKVDEEVVEEVADELVE 292
PfGA01_100041100       ELVEKVAEELVEKVDEQVAEELVE--------KVDEQVAEELVEKVDEQVVEEVADELVE 292
PfGN01_100041300       ELVEKVAEELVEKVDEQVAEELVE--------KVDEQVAEELVEKVDEQVVEEVADELVE 292
PfTG01_100041000       ELVEKVAEELVEKVDEQVAEELVE--------KVDEQVA--------------------- 271
PfDd2_100041100        ELVEKVAEELVEKVDEQVAEELVE--------KVDEQVAEE------------------- 273
PfSN01_100041200       ELVEKVAEELVEKVDEQVAEELVE--------KVDEQVAEELVEKV-------------- 278
PfCD01_100041000       ELVEKVAEELVEKVDEQVAEELVE--------KVDEQVAEELVEKVDEQVVEEVADELVE 292
PfKH02_100041200       ELVEKVAEELVEKVDEQVAE--------------------ELVEKVDEQVVEEVADELVE 280
Pf7G8_100040200        ELVEKVAEELVEKVDEQVAEELVE--------KVDEQVAEELVEKVDEQVVEEVADELVE 276
PfHB3_100040200        ELVEKVAEELVEKVDEQVAEELVE--------KVDEQVAEELVEKVDEQVVEEVADELVE 292
PfKH01_100040300       ELVEKVAEELVEKVDEQVAEELVE--------KVDEQVAEELVEKVDEQVVEEVADELVE 292
                       :: *:* **:.*:: *:**:

PF3D7_1035900          ------------------------------QKVDEEVTEELIEKVDEEVTEELIEKVDEE 325
PfSD01_100040400       KVDEEVVEKVPEEVVEEVAEEVAEEVVEEGEKVPEEVAEEVAEEVAEEVAEELVEKVDEQ 352
PfML01_100039900       KVVEEEGEKVPEEVVEEVAEEVAEEVVEEGEKVPEEVAEEVA----EEVAEELVEKVDEQ 348
PfKE01_100041100       KVVEEEGEKVPEEVVEEVAEEVAEEVVEEGEKVPEEVAEEVA----EEVAEELVEKVDEQ 348
PfIT_100039800         ------------------------------------------------------------ 266
PfGB4_100040700        KVVEEEGEKVPEEVVEEVAEEVAEEVVEEGEKVPEEVAEEVA----EEVAEELVEKVDEQ 348
PfGA01_100041100       KVVEEEGEKVPEEVVEEVAEEVAEEVVEEGEKVPEEVAEEVA----EEVAEELVEKVDEQ 348
PfGN01_100041300       KVVEEEGEKVPEEVVEEVAEEVAEEVVEEGEKVPEEVAEEVA----EEVAEELVEKVDEQ 348
PfTG01_100041000       ------------------------------------------------------------ 271
PfDd2_100041100        ------------------------------------------------------------ 273
PfSN01_100041200       --VEEEGEKVPEEVVEEVAEEVAEEVVEEGEKVPEEVAEEVA----EEVAEELVEKVDEQ 332
PfCD01_100041000       KVVEEEGEKVPEEVVEEVAEEVAEEVVEEGEKVPEEVAEEVA----EEVAEELVEKVDEQ 348
PfKH02_100041200       KVVEEEGEKVPEEVVEEVAEEVAEEVVEEGEKVPEEVAEEVA----EEVAEELVEKVDEQ 336
Pf7G8_100040200        KVDEEVVEK--------------------------------------------------- 285
PfHB3_100040200        KVVEEEGEKVPEEVVEEVAEEVAEEVVEEGEKVPEEVAEEVA----EEVAEELVEKVDEQ 348
PfKH01_100040300       KVVEEEGEKVPEEVVEEVAEEVAEEVVEEGEKVPEEVAEEVA----EEVAEELVEKVDEQ 348
```

Figure S1. Continued.

```
PF3D7_1035900          VAEELIEKVDEEVAEELIEKVADELVEKVAEELVEKVDEQVA--------EELVEKVDEQ 377
PfSD01_100040400       VAEELVEKVDEQVAEELVEKVDEQVVEEVADELVEKVDEEVVEKVDEEVVEEVADELVEK 412
PfML01_100039900       VAEELVEKVDEQVAEELVEKVDEQVVEEVADELVEKVDEEVVEKVDEEVVEEVADELVEK 408
PfKE01_100041100       VAEELVEKVDEQVAEELVEKVDEQVVEEVADELVEKVDEEVVEKVDEEVVEEVADELVEK 408
PfIT_100039800         -------------AEELVEKVDEQVVEEVADELVEKVDEELVEKVDEEVVEEVADELVEK 313
PfGB4_100040700        VAEELVEKVDEQVAEELVEKVDEQVVEEVADELVEKVDEEVVEKVDEEVVEEVADELVEK 408
PfGA01_100041100       VAEELVEKVDEQVAEELVEKVDEQVVEEVADELVEKVDEEVVEKVDEEVVEEVADELVEK 408
PfGN01_100041300       VAEELVEKVDEQVAEELVEKVDEQVVEEVADELVEKVDEEVVEKVDEEVVEEVADELVEK 408
PfTG01_100041000       --EELVEKVDEQVDEELVEKVDEQVVEEVADELVEKVDEEVVEKVDEEVVEEVADELVEK 329
PfDd2_100041100        ----LVEKVDEQVAEELVEKVDEQVVEEVADELVEKVDEEVVEKVDEEVVEEVADELVEK 329
PfSN01_100041200       VAEELVEKVDEQVAEELVEKVDEQVVEEVADELVEKVDEEVVEKVDEEVVEEVADELVEK 392
PfCD01_100041000       VAEELVEKVDEQVAEELVEKVDEQVVEEVADELVEKVDEEVVEKVDEEVVEEVADELVEK 408
PfKH02_100041200       VAEELVEKVDEQVAEELVEKVDEQVVEEVADELVEKVDEEVVEKVDEEVVEEVADELVEK 396
Pf7G8_100040200        ------------------------------------------------------------ 285
PfHB3_100040200        VAEELVEKVDEQVAEELVEKVDEQVVEEVADELVEKVDEEVVEKVDEEVVEEVADELVEK 408
PfKH01_100040300       VAEELVEKVDEQVAEELVEKVDEQVVEEVADELVEKVDEEVVEKVDEEVVEEVADELVEK 408


PF3D7_1035900          VAEELVEKVDEQVVEEVAEEVAEEVVEEGEKVPEEVAEEVAEE----------------- 420
PfSD01_100040400       VVEEEGEKVPEEVVEEVAE----------------------------------------- 431
PfML01_100039900       VVEEEGEKVPEEVVEEVAEEVAEEVVEEGEKVPEEVAEEVAEE----------------- 451
PfKE01_100041100       VVEEEGEKVPEEVVEEVAEEVAEEVVEEGEKVPEEVAEEVAE------------------ 450
PfIT_100039800         VVEEEGEKVPEEVAEEVAEEV--------------------------------------- 334
PfGB4_100040700        VVEEEGEKVPEEVVEEVAEEVAEEVVEEGEKVPEEVAEEVAEELVEKVDEQVAEELVEKV 468
PfGA01_100041100       VVEEEGEKVPEEVVEEVAEEVAEEVVEEGEKVPEEVAEEVAE------------------ 450
PfGN01_100041300       VVEEEGEKVPEEVVEEVAEEVAEEVVEEGEKVPEEVAEEVAEE----------------- 451
PfTG01_100041000       VVEEEGEKVPEEVVEEVAEEVAEEVVEEGEKVPEEVPEEVAEEV---------------- 373
PfDd2_100041100        VVEEEGEKVPEEVVEEVAEEVAEEVVEEGEKVPEEVAEEVAEEVAE-------------- 375
PfSN01_100041200       VVEEEGEKVPEEVVEEVAEEVAEEVVEEGEKVPEEVAEEVAEEVAEELVEKVDEQVAEEL 452
PfCD01_100041000       VVEEEGEKVPEEVVEEVAEEVAEEVVEEGEKVPEEVAEEVAEE----------------- 451
PfKH02_100041200       VVEEEGEKVPEEVVEEVAEEVAEEVVEEGEKVPEEV------------------------ 432
Pf7G8_100040200        ------------------------------------------------------------ 285
PfHB3_100040200        VVEEEGEKVPEEVVEEVAEEVAEEVVEEGEKVPEEVAEEVAE------------------ 450
PfKH01_100040300       VVEEEGEKVPEEVVEEVAEEVAEEVVEEGEKVPEEVAEEVAEE----------------- 451
```

Figure S1. Continued.

```
PF3D7_1035900        ------------------------------------------------------------ 420
PfSD01_100040400     ------------------------------------------------------------ 431
PfML01_100039900     ------------------------------------------------------------ 451
PfKE01_100041100     ------------------------------------------------------------ 450
PfIT_100039800       ------------------------------------------------------------ 334
PfGB4_100040700      ----DEQVAEELVEKVDEQVVEEVADELVEKVDEEVVEKVDEEVVEEVADELVEKVVEEE 524
PfGA01_100041100     ------------------------------------------------------------ 450
PfGN01_100041300     ------------------------------------------------------------ 451
PfTG01_100041000     --------------------AEEVAEELVEKVDEEVVEKVDEEVVEEVADELVEKVVEEE 413
PfDd2_100041100      --------------------------------------EVAEELVEKVDEEVAEKVVEEE 397
PfSN01_100041200     VEKVDEQVAEELVEKVDEQVVEEVADELVEKVDEEVVEKVDEEVVEEVADELVEKVVEEE 512
PfCD01_100041000     ------------------------------------------------------------ 451
PfKH02_100041200     ------------------------------------------------------------ 432
Pf7G8_100040200      ------------------------------------------------------------ 285
PfHB3_100040200      ------------------------------------------------------------ 450
PfKH01_100040300     ------------------------------------------------------------ 451

PF3D7_1035900        ------------------------------VAEEVAEEVAEELVEKVDEEVAEKVVEEEG 450
PfSD01_100040400     ---------------------------------EV----AEEL----------------- 437
PfML01_100039900     ------------------------------VAEEV----AEEL----------------- 460
PfKE01_100041100     ---------------------------------EV----AEEL----------------- 456
PfIT_100039800       ------------------------------------------------------------ 334
PfGB4_100040700      GEKVPEEVVEEVAEEVAEEVVEEGEKVPEEVAEEV----AEEL----------------- 563
PfGA01_100041100     ---------------------------------EV----AEEL----------------- 456
PfGN01_100041300     ------------------------------VAEEV----AEEL----------------- 460
PfTG01_100041000     GEKVPEEVVEEVAEEVAEEVVEEGEEVAEEVAEEVAEEVAEELVEKVDEEVAEKVVEEEG 473
PfDd2_100041100      GEKVLEEVIEEVAEEVAEEVVEEGEKVPEEVAEEVAEEVAEEVA---------------- 441
PfSN01_100041200     GEKVPEEVVEEVAEEVAEEVVEEGEKVPEEVAEEVAEEVAEEV----------------- 555
PfCD01_100041000     ------------------------------VAEEV----AEEL----------------- 460
PfKH02_100041200     -------------------------------AEEVAEEVAEE------------------ 443
Pf7G8_100040200      ------------------------------------------------------------ 285
PfHB3_100040200      ---------------------------------EV----AEEL----------------- 456
PfKH01_100040300     ------------------------------VAEEV----AEEL----------------- 460
```

Figure S1. Continued.

```
PF3D7_1035900         EKVPEEV----VEEVDEEVAEKVVEEEGEKVLEEVIEEVVEEVAEEVAEKVVEEQGEKVN 506
PfSD01_100040400      -----------VEKVDEEVAEKVVEEEGEKVLEEVIEEVVEEVAEEVAEKVVEEQGEKVN 486
PfML01_100039900      -----------VEKVDEEVAEKVVEEEGEKVLEEVIEEVVEEVAEEVAEKVVEEQGEKVN 509
PfKE01_100041100      -----------VEKVDEEVAEKVVEEEGEKVLEEVIEEVVEEVAEEVAEKVVEEQGEKVN 505
PfIT_100039800        -------AEELVEKVDEEVAEKVVEEEGEKVLEEVIEEVVEEVAEEVAEKVVEEQGEKVN 387
PfGB4_100040700       -----------VEKVDEEVAEKVVEEEGEKVLEEVIEEVVEEVAEEVAEKVVEEQGEKVN 612
PfGA01_100041100      -----------VEKVDEEVAEKVVEEEGEKVLEEVIEEVVEEVAEEVAEKVVEEQGEKVN 505
PfGN01_100041300      -----------VEKVDEEVAEKVVEEEGEKVLEEVIEEVVEEVAEEVAEKVVEEQGEKVN 509
PfTG01_100041000      EKVLEEVIEEVVEKVDEEVAEKVVEEEGEKVLEEVIEEVVEEVAEEVAEKVVEEQGEKVN 533
PfDd2_100041100       ----EEVAEELVEKVDEEVAEKVVEEEGEKVLEEVIEEVVEEVAEEVAEKVVEEQGEKVN 497
PfSN01_100041200      -------AEELVEKVDEEVAEKVVEEEGEKVLEEVIEEVVEEVAEEVAEKVVEEQGEKVN 608
PfCD01_100041000      -----------VEKVDEEVAEKVVEEEGEKVLEEVIEEVVEEVAEEVAEKVVEEQGEKVN 509
PfKH02_100041200      ------VAEELVEKVDEEVAEKVVEEEGEKVLEEVIEEVVEEVAEEVAEKVVEEQGEKVN 497
Pf7G8_100040200       ----------------------------------VVEEVVEEVAEEVAEKVVEEQGEKVN 311
PfHB3_100040200       -----------VEKVDEEVAEKVVEEEGEKVLEEVIEEVVEEVAEEVAEKVVEEQGEKVN 505
PfKH01_100040300      -----------VEKVDEEVAEKVVEEEGEKVLEEVIEEVVEEVAEEVAEKVVEEQGEKVN 509
                                                        *:************************

PF3D7_1035900         KNDLNDASSEEIKDSSDFKESHEELFKVFLELINKNDLVKENLKKITNNLNEMHLSTLYP 566
PfSD01_100040400      KNDLNDASSEEIKDSSDFKESHEELFKVFLELINKNDLVKENLKKITNNLNEMHLSTLYP 546
PfML01_100039900      KNDLNDASSEEIKDSSDFKESHEELFKVFLELINKNDLVKENLKKITNNLNEMHLSTLYP 569
PfKE01_100041100      KNDLNDASSEEIKDSSDFKESHEELFKVFLELINKNDLVKENLKKITNNLNEMHLSTLYP 565
PfIT_100039800        KNDLNDASSEEIKDSSDFKESHEELFKVFLELINKNDLVKENLKKITNNLNEMHLSTLYP 447
PfGB4_100040700       KNDLNDASSEEIKDSSDFKESHEELFKVFLELINKNDLVKENLKKITNNLNEMHLSTLYP 672
PfGA01_100041100      KNDLNDASSEEIKDSSDFKESHEELFKVFLELINKNDLVKENLKKITNNLNEMHLSTLYP 565
PfGN01_100041300      KNDLNDASSEEIKDSSDFKESHEELFKVFLELINKNDLVKENLKKITNNLNEMHLSTLYP 569
PfTG01_100041000      KNDLNDASSEEIKDSSDFKESHEELFKVFLELINKNDLVKENLKKITNNLNEMHLSTLYP 593
PfDd2_100041100       KNDLNDASSEEIKDSSDFKESHEELFKVFLELINKNDLVKENLKKITNNLNEMHLSTLYP 557
PfSN01_100041200      KNDLNDASSEEIKDSSDFKESHEELFKVFLELINKNDLVKENLKKITNNLNEMHLSTLYP 668
PfCD01_100041000      KNDLNDASSEEIKDSSDFKESHEELFKVFLELINKNDLVKENLKKITNNLNEMHLSTLYP 569
PfKH02_100041200      KNDLNDASSEEIKDSSDFKESHEELFKVFLELINKNDLVKENLKKITNNLNEMHLSTLYP 557
Pf7G8_100040200       KNDLNDASSEEIKDSSDFKESHEELFKVFLELINKNDLVKENLKKITNNLNEMHLSTLYP 371
PfHB3_100040200       KNDLNDASSEEIKDSSDFKESHEELFKVFLELINKNDLVKENLKKITNNLNEMHLSTLYP 565
PfKH01_100040300      KNDLNDASSEEIKDSSDFKESHEELFKVFLELINKNDLVKENLKKITNNLNEMHLSTLYP 569
                      ************************************************************
```


Reference:

Sievers F, Wilm A, Dineen D, Gibson TJ, Karplus K, Li W, Lopez R, McWilliam H, Remmert M, Söding J, Thompson JD, Higgins DG. Fast, scalable generation of high-quality protein multiple sequence alignments using Clustal Omega. Mol Syst Biol. 2011 Oct 11;7:539. doi: 10.1038/msb.2011.75.
