# Supplementary material for: Plasmodium falciparum Merozoite Associated Armadillo Protein (PfMAAP) Is Apically Localized in Free Merozoites and Antibodies Are Associated With Reduced Risk of Malaria
Source: Front Immunol. 2020 Apr 7;11:505. doi: 10.3389/fimmu.2020.00505 (PMC7155890; doi:10.3389/fimmu.2020.00505)
Supplement: Figure S2 — Amino acid alignment of full-length sequences from non-malaria isolates for PfMAAP (PF3D7_1035900) aligned with the 3D7 isolate as a reference. Alignments were generated using Clustal Omega (Sievers et al., 2011). Plasmodium falciparum, PF3D7_1035900; Plasmodium rechenowi, PRG01_1034400 and PRCDC_1035200; Plasmodium billcolllinsi, PBILCG01_1034800; Plasmodium gaboni, PGSY75_0012400; Plasmodium praefalciparum, PPRFG01_1036900; and Plasmodium adleri, PADL01_1034600. All sequences were obtained from Plasmodb (https://plasmodb.org/plasmo/). [file Image_2.pdf]

Figure S2. Amino acid alignment of full-length sequences from non-malaria isolates for PfMAAP (PF3D7_1035900) aligned with the 3D7 isolate as a reference. Alignments were generated using Clustal Omega (Sievers et al., 2011). *Plasmodium falciparum*, PF3D7_1035900; *P.rechenowi*, PRG01_1034400 and PRCDC_1035200; *P.billcolllinsi*, PBILCG01_1034800; *P.gaboni*, PGSY75_0012400; *P.praefalciparum*, PPRFG01_1036900; and *P.adleri*, PADL01_1034600. All sequences were obtained from Plasmodb (https://plasmodb.org/plasmo/).

```
PF3D7_1035900           MLNIFNIIFLLFLINIYICEANGTLSENIESAEEIDALKTNLRNGYLNNTYFNEENNNLN 60
PRG01_1034400           MLNIFNIIFLLFLINIYICEANGALSENIESAEEIDTLKTNLRNGYLNNTYFNEENNNLN 60
PRCDC_1035200           MLNIFNIIFLLFLINIYICEANGALSENIESAEEIDTLKTNLRNGYLNNTYFNEENNNLN 60
PBILCG01_1034800        MMNIFYIIFLLFLINIYTCETIGALSENVESVEEIDTLKTNLRNGNLNNTYFNEENNNLN 60
PGSY75_0012400          MMNFFYIIFLLFLINLYICETYGALSENIESAEEIDTLKTSLRNGHLNNTYFNEENNNLN 60
PPRFG01_1036900         MLNIFNIIFLLFLINIYICEANGTLSENIESAEEIDALKTNLRNGYLNNTYFNEENNNLN 60
PADL01_1034600          MMNFFYIIFLLFLINLYICETNGALSENIESAEEIDTLKTSLRNGYLNNTYFNEEKNNLN 60
                        *:*:* *********:* **: *:****:**.****:***.**** *********:****

PF3D7_1035900           IENEINNTNYNEVTEETKEELYDINENIFPDYFFLDIFTENKEQKNEEVPMKIEVVNDGE 120
PRG01_1034400           IGNEINNTNYNEVTEETKEELYDINQNIFPDYFFLDIVPEKKEQKNEEVPMKIEVVNDGE 120
PRCDC_1035200           IGNEINNTNYNEVTEETKEELYDINQNIFPDYFFLDIVPENKEQKNEEVPMKIEVVNDGE 120
PBILCG01_1034800        IGNEINNTNYNEVTEETKEELYDINENIFPEYFFLDIVTENKEQKNEEVSMKTEVVNDGE 120
PGSY75_0012400          IGNEINNTNYKEVTEESKEELYDINENIFPDYFFLDIVTENQEQKNEEVPVKTEVVSDEE 120
PPRFG01_1036900         IENEINNTNYNEVTEETKEELYDINENIFPDYFFLDIFTENKEQKNEEVPMKIEVVNDGE 120
PADL01_1034600          IGNEINNTNYNEVTQESKEELFDINENISPDYFFLDIVTENKEQKNEEVPVKTEVVSNEE 120
                        * ********:***:*:****:***:** *:******. *::******* :* ***.: *

PF3D7_1035900           EVKTEYVSEKNEEVENKSETEIGEELTEKVDEKVPEEVAEEL--------VEKVDEEVAE 172
PRG01_1034400           EVKTESATEKNEEVENKSATEIGEEVVEKVDEKVDEKVDEKVDEEVVEELVEEEGEEVAE 180
PRCDC_1035200           EVKTESATEKNEEVENKSATEIGEEVDEKVDEKVDEKVDEKVDGEV----VEEVAEEVAD 176
PBILCG01_1034800        EVGDESIIEE-E-VETESITEENEEVETESVTEEN----EEVETES----VIEIAEKVP- 169
PGSY75_0012400          ELETESVIEEVE-VETESINDEG-------------------ETES----VIEEIEIET- 155
PPRFG01_1036900         EVKTEYVSEKNEEVENKSETEIGEELTEKVDEIVDEKVPEEVAEVVEE--VPEEVDEVTE 178
PADL01_1034600          ELETESVIEEVE-VETESISDEE--VETESISD------EEVETES----VTEEVEVET- 166
                        *:  *   *: * **.:* .:                             * :  :

PF3D7_1035900           ELVEKVDEKVAEEVDQKVDEEVTEELIEKVDEEVTEELIEKVDEEVAEELIEKVDEEVAE 232
PRG01_1034400           ELIEKVDEE----VADEVAEEVAEEVAEEIDEEVVEELIEKVDEKVPEEVVEEVVEEVAD 236
PRCDC_1035200           EVA------------EEVADEVAEEVVQTVDEEVVEELIEKVDEKVDEEVADEIDEEVAE 224
PBILCG01_1034800        ---------------E--------------------------------EVVEEVAEEVPE 182
PGSY75_0012400          ---------------ESIND-----------EGET------------ETVIEEIEIETES 177
PPRFG01_1036900         ELI------------EKVDEEVTEELIEKVDEKVTEELIEKVDEKVPEEVADEVVEEVVE 226
PADL01_1034600          ---------------ESIND-----------EGET------------ESVIEEVEVETES 188
                                       :                                 : :::  *. .
```

Figure S2. Continued

```
PF3D7_1035900         ELIEKVAD------------------ELIEKVDEEVAEE--------LIEKVADELVEKV 266
PRG01_1034400         EVADGVAEEID----------------------------------EEVVEEVVEEVADEV 262
PRCDC_1035200         EMDEEVVEELIEKVDESVAEV---V--------------------EEVVEEVAEEVVEEE 261
PBILCG01_1034800      VV-----EEVVEEVAEEVPEV---V--------------------EEVVEEVAEEVVEEV 214
PGSY75_0012400        INDEGETETVIEEIEIETESI---N--------------------SS-------DL---K 204
PPRFG01_1036900       EVADELVEKVVEEVVEEVDEVAEVAEEVDQKVDEEVTEELIEKVDEKVDEEVAEELIEKV 286
PADL01_1034600        INDEGETESVIEEIEIETESI---N--------------------DE-------EG---E 215
                             :                                              :

PF3D7_1035900         AEELVEKVDEEVAEELVEKVDEKVAEEVDQKVDEEVTEELIEKVDEEVTEELIEKVDEEV 326
PRG01_1034400         AEEVVEEVADEVAEELIEKVDEEVADEVAEEVAEEVAEEIDE----------------EV 306
PRCDC_1035200         GEKLVEEEGEEVAEELIEKVDEEVAEEVAEEVAEEVV----------------------- 298
PBILCG01_1034800      ------------------------------------------------------------ 214
PGSY75_0012400        ------------------------------------------------------------ 204
PPRFG01_1036900       AEELVEKVDEKVADELVEKVDEEVAEEVVEEVAEEVAEELIEKVDEEVVEKVDEEVAEEL 346
PADL01_1034600        ------------------------------------------------------------ 215

PF3D7_1035900         AEELIEKVDEEVAEELIEKVADELVEKVAEELVEKV----------------DEQVA--- 367
PRG01_1034400         VEELIEKVDEKVPEEVV----EEVVEEVADEVADGVAEEIDEEVVEELIEKVDEKVPEEV 362
PRCDC_1035200         -----QTVDEEVVEELIEKVDEKVDEEVADEVADGVAEEIDEEVVEELIEKVDEKVPEEV 353
PBILCG01_1034800      --------VEEVVEEVVEEVPEKMPE----EVPEKVSEEVP-----E-KVSEE--VPEKV 254
PGSY75_0012400        --------ESH------------------------------------------------- 207
PPRFG01_1036900       VEELIEKVDEEVAEELIEKVDEEVVEEVADELVEKVDEEVVEEVADELVEKVDEEVVEEV 406
PADL01_1034600        --------TESVIEEIEIE-TESINDE---GETESVIEEIEIET--E-SINDEEGETESV 260
                               .

PF3D7_1035900         -EELVEKVDEQVAEELV--------EKVDEQVVEEVAEEVAEEVVEEGEKVPEEVAEEVA 418
PRG01_1034400         VEEVVEEVDEEVDEKVP--------EEVVEEVADEV-----------ADGVAEEVVEEVV 403
PRCDC_1035200         VEEVVEEVDEKVPEEVVE---------VVEEVADEV-----------ADGVAEEVVEEVV 393
PBILCG01_1034800      SEEVPEEVHEEAPEEVV---------------------------------------EEVA 275
PGSY75_0012400        ------------------------------------------------------------ 207
PPRFG01_1036900       ADELVEKVDEEVAEEVVEEEGEKVAEEVVEEVAEEVAEEVAEEVVEEGEKVPEEVVEEVA 466
PADL01_1034600        IEEIEIETESINDEEVE------------------------------------------T 278

PF3D7_1035900         EEVAEEVAEEVAEELV----------------EKVDEEVAEKVVEEEGEKVPEEVVEEVD 462
PRG01_1034400         EELIEQVDEKVAEEVVEEVVEEVAEKLVEEEGEKVPEEVAEKVVQEEGEKVPEEVVEEVA 463
PRCDC_1035200         EELIEQVDEKVAEEVVEEVVEEVAEKLVEEEGEKVPEEVAEKVVQEEGEKVPEEVVEEVA 453
PBILCG01_1034800      EKIPEA---------------------VEDAAEKIPEAV-----EDVAEKIPE-VVEDVA 308
PGSY75_0012400        ------------------------------------------------------------ 207
PPRFG01_1036900       EELVEKVDEEVAEEVAEEVAEEVAEELVEKVDEEVVEEVAEKVVEEEGEKVLEEVI---- 522
PADL01_1034600        ESVIEE---------------------IEIETESINDEE-----EVETESVTE-EVE--- 308
```

Figure S2. Continued

```
PF3D7_1035900         EEVAEKVVE----EEGEKVLEEVIEEVVEEVAEEVAEKVVEEQGEKVNKNDLNDASSEEI 518
PRG01_1034400         EEVAEEVAEEVAEEVAEKVVEEVAEEVAEKVVEEVAEKVVEEEGEKVNKNDLNDAASEEI 523
PRCDC_1035200         EEVAEEVAE----------------EVAEKVVEEVAEKVIEEEGEKVNKNDLNDAASEEI 497
PBILCG01_1034800      EE----VVE----------------EVAEKIPEV--VEDVAEKVEKINKNDLNDAASEEI 346
PGSY75_0012400        ------------------------------------------------------------ 207
PPRFG01_1036900       --------E----------------EVVEEVAEEVAEKVVEEEGEKVNKNDLNDACF--- 555
PADL01_1034600        ------VET----------------ESINDEEGE--TESVAAVVEKVNKNDLNDAASEEI 344

PF3D7_1035900         KDSSDFKESHEELFKVFLELINKNDLVKENLKKITNNLNEMHLSTLYP     566
PRG01_1034400         KDSSDFKESHEELFKVFMELINKNDLVKENLKKLTNTLNEMNISTLYP     571
PRCDC_1035200         KDSSDFKESHEELFKVFMELINKNDLVKENLKKLTNTLNEMNISTLYP     545
PBILCG01_1034800      KDSSDFKESHEELFKVFLELIDKNDLVKENLKKLTKILNEINLSTIYP     394
PGSY75_0012400        ------------------------------------------------     207
PPRFG01_1036900       -RASDFKESHEELFKVFLELINKNDLVKENLKKLTNNLNEMHLSTLYP     602
PADL01_1034600        KDSSDFKESH--------------------------------------     354
                      *  ******************:************** ***  :**:**
```

Reference:

Sievers F, Wilm A, Dineen D, Gibson TJ, Karplus K, Li W, Lopez R, McWilliam H, Remmert M, Söding J, Thompson JD, Higgins DG. Fast, scalable generation of high-quality protein multiple sequence alignments using Clustal Omega. Mol Syst Biol. 2011 Oct 11;7:539. doi: 10.1038/msb.2011.75.
