# Supplementary material for: Plasmodium falciparum Merozoite Associated Armadillo Protein (PfMAAP) Is Apically Localized in Free Merozoites and Antibodies Are Associated With Reduced Risk of Malaria
Source: Front Immunol. 2020 Apr 7;11:505. doi: 10.3389/fimmu.2020.00505 (PMC7155890; doi:10.3389/fimmu.2020.00505)
Supplement: Figure S3 — N- and C-terminal semi-conserved and armadillo repeat sequences for PfMAAP in (A) sixteen laboratory adapted isolates (PF3D7_1035900, PfSD01_100040400, PfML01_100039900, PfKE01_100041100, PfIT_100039800, PfGB4_100040700, PfGN01_100041300, PfTG01_100041000, PfDd2_100041100, PfSN01_100041200, PfKH02_100041200, Pf7G8_100040200, PfHB3_100040200, PfGA01_100041100, PfKH01_100040300, PfCD01_100041000) and (B) in five Laverania subgenus of Plasmodium (P.rechenowi, PRG01_1034400 and PRCDC_1035200; P.billcolllinsi, PBILCG01_1034800; P. gaboni, PGSY75_0012400; P.praefalciparum, PPRFG01_1036900; and P. adleri, PADL01_1034600. All sequences were obtained from Plasmodb (https://plasmodb.org/plasmo/). [file Image_3.pdf]

Supplementary Figure S3A. N- and C-terminal semi-conserved and armadillo repeat sequences for *Pf*MAAP in sixteen laboratory adapted isolates (PF3D7_1035900, PfSD01_100040400, PfML01_100039900, PfKE01_100041100, PfIT_100039800, PfGB4_100040700, PfGN01_100041300, PfTG01_100041000, PfDd2_100041100, PfSN01_100041200, PfKH02_100041200, Pf7G8_100040200, PfHB3_100040200, PfGA01_100041100, PfKH01_100040300, PfCD01_100041000)

Plasmodium falciparum, 3D7 isolate

N-terminal

MLNIFNIIFLLFLINIYICEANGTLSENIESAEEIDALKTNLRNGYLNNTYFNEENNNLNIENEINNTNYNEVTEETKEELYDINENIFPDYFFLDIFTENKEQKNEEVPMKIEVVNDGEEVKTEYVSEKNEEVENKSETEIG

Central Repeat

EELTEKVDEKVPEEVAEELVEKVDEEVAEELVEKVDEKVAEEVDQKVDEEVTEELIEKVDEEVTEELIEKVDEEVAEELIEKVDEEVAEELIEKVADELIEKVDEEVAEELIEKVADELVEKVAEELVEKVDEEVAEELVEKV
DEKVAEEVDQKVDEEVTEELIEKVDEEVTEELIEKVDEEVAEELIEKVDEEVAEELIEKVADELVEKVAEELVEKVDEQVAEELVEKVDEQVAEELVEKVDEQVVEEVAEEVAEEVVEEGEKVPEEVAEEVAEEVAEEV
AEELVEKVDEEVAEKVVEEEGEKVPEEVVEEVDEEVAEKVVEEEGEKVLEEVIEEVVEEVAEEVAEKVVEEQGEK

C-terminal

VNKNDLNDASSEEIKDSSDFKESHEELFKVFLELINKNDLVKENLKKITNNLNEMHLSTLYP

P. falciparum, 7G8 isolate (371)

N-terminal

MLNIFNIIFLLFLINIYICEANGTLSENIESAEEIDALKTNLRNGYLNNTYFNEENNNLNIENEINNTNYNEVTEETKEELYDINENIFPDYFFLDIFTENKEQKNEEVPMKIEVVNDGEEVKTEYVSEKNEEVENKSETEIG

Central Repeat

EELTEKVDEKVPEEVAEELVEKVDEEVAEELVEKVDEKVDQKVDEEVTEELIEKVDEEVTEELIEKVDEEVAEELIEKVADELVEKVAEELVEKVDEQVAEELVEKVDEQVAEELVEKVDEQVVEEVADELVEKVDEEVVEKV
VEEVVEEVAEEVAEKVVEEQGEK

C-terminal

VNKNDLNDASSEEIKDSSDFKESHEELFKVFLELINKNDLVKENLKKITNNLNEMHLSTLYP

Supplementary Figure S3A. continued

P. falciparum, CD01 isolate (569)

N-terminal

MLNIFNIIFLLFLINIYICEANGTLSENIESAEEIDALKTNLRNGYLNNTYFNEENNNLNIENEINNTNYNEVTEETKEELYDINENIFPDYFFLDIFTENKEQKNEEVPMKIEVVNDGEEVKTEYVSEKNEEVENKSETEIG

Central Repeat

EELTEKVDEKVPEEVAEELVEKVDEEVAEELVEKVDEKVAEEVDQKVDEEVTEELIEKVDEEVTEELIEKVDEEVAEELIEKVDEEVAEELIEKVADELVEKVAEELVEKVDEQVAEELVEKVDEQVAEELVEKVDEQVVEEVAD
ELVEKVVEEEGEKVPEEVVEEVAEEVAEEVVEEGEKVPEEVAEEVAEEVAEELVEKVDEQVAEELVEKVDEQVAEELVEKVDEQVVEEVADELVEKVDEEVVEKVDEEVVEEVADELVEKVVEEEGEKVPEEVVEEVAEEVAEEV
VEEGEKVPEEVAEEVAEEVAEEVAEELVEKVDEEVAEKVVEEEGEKVLEEVIEEVVEEVAEEVAEKVVEEQGEK

C-terminal

VNKNDLNDASSEEIKDSSDFKESHEELFKVFLELINKNDLVKENLKKITNNLNEMHLSTLYP

P. falciparum, Dd2 isolate (557)

N-terminal

MLNIFNIIFLLFLINIYICEANGTLSENIESAEEIDALKTNLRNGYLNNTYFNEENNNLNIENEINNTNYNEVTEETKEELYDINENIFPDYFFLDIFTENKEQKNEEVPMKIEVVNDGEEVKTEYVSEKNEEVENKSETEIG

Central Repeat

EELTEKVDEKVPEEVAEELVEKVDEEVAEELVEKVDEKVAEEVDQKVDEEVTEELIEKVDEEVTEELIEKVDEEVAEELIEKVDEEVAEELIEKVADELVEKVAEELVEKVDEQVAEELVEKVDEQVAEELVEKVDEQVAEEL
VEKVDEQVVEEVADELVEKVDEEVVEKVDEEVVEEVADELVEKVVEEEGEKVPEEVVEEVAEEVAEEVVEEGEKVPEEVAEEVAEEVAEEVAEELVEKVDEEVAEKVVEEEGEKVLEEVIEEVAEEVAEEVVEEGEKVPEEVA
EEVAEEVAEEVAEEVAEELVEKVDEEVAEKVVEEEGEKVLEEVIEEVVEEVAEEVAEKVVEEQGEK

C-terminal

VNKNDLNDASSEEIKDSSDFKESHEELFKVFLELINKNDLVKENLKKITNNLNEMHLSTLYP

P. falciparum, GA01 isolate (565)

N-terminal

MLNIFNIIFLLFLINIYICEANGTLSENIESAEEIDALKTNLRNGYLNNTYFNEENNNLNIENEINNTNYNEVTEETKEELYDINENIFPDYFFLDIFTENKEQKNEEVPMKIEVVNDGEEVKTEYVSEKNEEVENKSETEIG

Central Repeat

EELTEKVDEKVPEEVAEELVEKVDEEVAEELVEKVDEKVAEEVDQKVDEEVTEELIEKVDEEVTEELIEKVDEEVAEELIEKVDEEVAEELIEKVADELVEKVAEELVEKVDEQVAEELVEKVDEQVAEELVEKVDEQVVEEV
ADELVEKVVEEEGEKVPEEVVEEVAEEVAEEVVEEGEKVPEEVAEEVAEEVAEELVEKVDEQVAEELVEKVDEQVAEELVEKVDEQVVEEVADELVEKVDEEVVEKVDEEVVEEVADELVEKVVEEEGEKVPEEVVEEVAEEV
AEEVVEEGEKVPEEVAEEVAEEVAEELVEKVDEEVAEKVVEEEGEKVLEEVIEEVVEEVAEEVAEKVVEEQGEK

C-terminal

VNKNDLNDASSEEIKDSSDFKESHEELFKVFLELINKNDLVKENLKKITNNLNEMHLSTLYP

Supplementary Figure S3A. continued

P. falciparum, GB4 isolate (672)

N-terminal

MLNIFNIIFLLFLINIYICEANGTLSENIESAEEIDALKTNLRNGYLNNTYFNEENNNLNIENEINNTNYNEVTEETKEELYDINENIFPDYFFLDIFTENKEQKNEEVPMKIEVVNDGEEVKTEYVSEKNEEVENKSETEIG

Central Repeat

EELTEKVDEKVPEEVAEELVEKVDEEVAEELVEKVDEKVAEEVDQKVDEEVTEELIEKVDEEVTEELIEKVDEEVAEELIEKVDEEVAEELIEKVADELVEKVAEELVEKVDEQVAEELVEKVDEQVAEELVEKVDEEVVEEV
ADELVEKVVEEEGEKVPEEVVEEVAEEVAEEVVEEGEKVPEEVAEEVAEEVAEELVEKVDEQVAEELVEKVDEQVAEELVEKVDEQVVEEVADELVEKVDEEVVEKVDEEVVEEVADELVEKVVEEEGEKVPEEVVEEVAEEV
AEEVVEEGEKVPEEVAEEVAEELVEKVDEQVAEELVEKVDEQVAEELVEKVDEQVVEEVADELVEKVDEEVVEKVDEEVVEEVADELVEKVVEEEGEKVPEEVVEEVAEEVAEEVVEEGEKVPEEVAEEVAEELVEKVDEEVA
EKVVEEEGEKVLEEVIEEVVEEVAEEVAEKVVEEQGEK

C-terminal

VNKNDLNDASSEEIKDSSDFKESHEELFKVFLELINKNDLVKENLKKITNNLNEMHLSTLYP

P. falciparum, GN01 isolate (569)

N-terminal

MLNIFNIIFLLFLINIYICEANGTLSENIESAEEIDALKTNLRNGYLNNTYFNEENNNLNIENEINNTNYNEVTEETKEELYDINENIFPDYFFLDIFTENKEQKNEEVPMKIEVVNDGEEVKTEYVSEKNEEVENKSETEIG

Central Repeat

EELTEKVDEKVPEEVAEELVEKVDEEVAEELVEKVDEKVAEEVDQKVDEEVTEELIEKVDEEVTEELIEKVDEEVAEELIEKVDEEVAEELIEKVADELVEKVAEELVEKVDEQVAEELVEKVDEQVAEELVEKVDEQVVEEV
ADELVEKVVEEEGEKVPEEVVEEVAEEVAEEVVEEGEKVPEEVAEEVAEEVAEELVEKVDEQVAEELVEKVDEQVAEELVEKVDEQVVEEVADELVEKVDEEVVEKVDEEVVEEVADELVEKVVEEEGEKVPEEVVEEVAEEV
AEEVVEEGEKVPEEVAEEVAEEVAEEVAEELVEKVDEEVAEKVVEEEGEKVLEEVIEEVVEEVAEEVAEKVVEEQGEK

C-terminal

VNKNDLNDASSEEIKDSSDFKESHEELFKVFLELINKNDLVKENLKKITNNLNEMHLSTLYP

P. falciparum, HB3 isolate (565)

N-terminal

MLNIFNIIFLLFLINIYICEANGTLSENIESAEEIDALKTNLRNGYLNNTYFNEENNNLNIENEINNTNYNEVTEETKEELYDINENIFPDYFFLDIFTENKEQKNEEVPMKIEVVNDGEEVKTEYVSEKNEEVENKSETEIG

Central Repeat

EELTEKVDEKVPEEVAEELVEKVDEEVAEELVEKVDEKVAEEVDQKVDEEVTEELIEKVDEEVTEELIEKVDEEVAEELIEKVDEEVAEELIEKVADELVEKVAEELVEKVDEQVAEELVEKVDEQVAEELVEKVDEQVVEEV
ADELVEKVVEEEGEKVPEEVVEEVAEEVAEEVVEEGEKVPEEVAEEVAEEVAEELVEKVDEQVAEELVEKVDEQVAEELVEKVDEQVVEEVADELVEKVDEEVVEKVDEEVVEEVADELVEKVVEEEGEKVPEEVVEEVAEEV
AEEVVEEGEKVPEEVAEEVAEEVAEELVEKVDEEVAEKVVEEEGEKVLEEVIEEVVEEVAEEVAEKVVEEQGEK

C-terminal

VNKNDLNDASSEEIKDSSDFKESHEELFKVFLELINKNDLVKENLKKITNNLNEMHLSTLYP

Supplementary Figure S3A. continued

P. falciparum, IT isolate (447)

N-terminal

MLNIFNIIFLLFLINIYICEANGTLSENIESAEEIDALKTNLRNGYLNNTYFNEENNNLNIENEINNTNYNEVTEETKEELYDINENIFPDYFFLDIFTENKEQKNEEVPMKIEVVNDGEEVKTEYVSEKNEEVENKSETEIG

Central Repeat

EELTEKVDEKVPEEVAEELVEKVDEEVAEELVEKVDEKVDQKVDEEVTEELIEKVDEEVTEELIEKVDEEVAEELIEKVDEEVAEELIEKVADELVEKVAEELVEKVDEQVAEELVEKVDEQVAEELVEKVDEQVVEEVADEL
VEKVDEELVEKVDEEVVEEVADELVEKVVEEEGEKVPEEVAEEVAEEVAEELVEKVDEEVAEKVVEEEGEKVLEEVIEEVVEEVAEEVAEKVVEEQGEK

C-terminal

VNKNDLNDASSEEIKDSSDFKESHEELFKVFLELINKNDLVKENLKKITNNLNEMHLSTLYP

P. falciparum, KE01 isolate (565)

N-terminal

MLNIFNIIFLLFLINIYICEANGTLSENIESAEEIDALKTNLRNGYLNNTYFNEENNNLNIENEINNTNYNEVTEETKEELYDINENIFPDYFFLDIFTENKEQKNEEVPMKIEVVNDGEEVKTEYVSEKNEEVENKSETEIG

Central Repeat

EELTEKVDEKVPEEVAEELVEKVDEEVAEELVEKVDEKVAEEVDQKVDEEVTEELIEKVDEEVTEELIEKVDEEVAEELIEKVADELVEKVAEELVEKVDEQVAEELVEKVDEQVAEELVEKVDEQVVEEV
ADELVEKVVEEEGEKVPEEVVEEVAEEVAEEVVEEGEKVPEEVAEEVAEEVAEELVEKVDEQVAEELVEKVDEQVAEELVEKVDEQVVEEVADELVEKVDEEVVEKVDEEVVEEVADELVEKVVEEEGEKVPEEVVEEVAEEV
AEEVVEEGEKVPEEVAEEVAEEVAEELVEKVDEEVAEKVVEEEGEKVLEEVIEEVVEEVAEEVAEKVVEEQGEK

C-terminal

VNKNDLNDASSEEIKDSSDFKESHEELFKVFLELINKNDLVKENLKKITNNLNEMHLSTLYP

P. falciparum, KH01 isolate (569)

N-terminal

MLNIFNIIFLLFLINIYICEANGTLSENIESAEEIDALKTNLRNGYLNNTYFNEENNNLNIENEINNTNYNEVTEETKEELYDINENIFPDYFFLDIFTENKEQKNEEVPMKIEVVNDGEEVKTEYVSEKNEEVENKSETEIG

Central Repeat

EELTEKVDEKVPEEVAEELVEKVDEEVAEELVEKVDEKVAEEVDQKVDEEVTEELIEKVDEEVTEELIEKVDEEVAEELIEKVDEEVAEELIEKVADELVEKVAEELVEKVDEQVAEELVEKVDEQVAEELVEKVDEQVVEEV
ADELVEKVVEEEGEKVPEEVVEEVAEEVAEEVVEEGEKVPEEVAEEVAEEVAEELVEKVDEQVAEELVEKVDEQVAEELVEKVDEQVVEEVADELVEKVDEEVVEKVDEEVVEEVADELVEKVVEEEGEKVPEEVVEEVAEEV
AEEVVEEGEKVPEEVAEEVAEEVAEEVAEELVEKVDEEVAEKVVEEEGEKVLEEVIEEVVEEVAEEVAEKVVEEQGEK

C-terminal

VNKNDLNDASSEEIKDSSDFKESHEELFKVFLELINKNDLVKENLKKITNNLNEMHLSTLYP

Supplementary Figure S3A. continued

P. falciparum, KH02 isolate (557)

N-terminal

MLNIFNIIFLLFLINIYICEANGTLSENIESAEEIDALKTNLRNGYLNNTYFNEENNNLNIENEINNTNYNEVTEETKEELYDINENIFPDYFFLDIFTENKEQKNEEVPMKIEVVNDGEEVKTEYVSEKNEEVENKSETEIG

Central Repeat

EELTEKVDEKVPEEVAEELVEKVDEEVAEELVEKVDEKVAEEVDQKVDEEVTEELIEKVDEEVTEELIEKVDEEVAEELIEKVDEEVAEELIEKVADELVEKVAEELVEKVDEQVAEELVEKVDEQVVEEVADELVEKVVEEE
GEKVPEEVVEEVAEEVAEEVVEEGEKVPEEVAEEVAEEVAEELVEKVDEQVAEELVEKVDEQVAEELVEKVDEQVVEEVADELVEKVDEEVVEKVDEEVVEEVADELVEKVVEEEGEKVPEEVVEEVAEEVAEEVVEEGEKVP
EEVAEEVAEEVAEEVAEELVEKVDEEVAEKVVEEEGEKVLEEVIEEVVEEVAEEVAEKVVEEQGEK

C-terminal

VNKNDLNDASSEEIKDSSDFKESHEELFKVFLELINKNDLVKENLKKITNNLNEMHLSTLYP

P. falciparum, ML01 isolate (569)

N-terminal

MLNIFNIIFLLFLINIYICEANGTLSENIESAEEIDALKTNLRNGYLNNTYFNEENNNLNIENEINNTNYNEVTEETKEELYDINENIFPDYFFLDIFTENKEQKNEEVPMKIEVVNDGEEVKTEYVSEKNEEVENKSETEIG

Central Repeat

EELTEKVDEKVPEEVAEELVEKVDEEVAEELVEKVDEKVAEEVDQKVDEEVTEELIEKVDEEVTEELIEKVDEEVAEELIEKVDEEVAEELIEKVADELVEKVAEELVEKVDEQVAEELVEKVDEQVAEELVEKVDEQVVEEV
ADELVEKVVEEEGEKVPEEVVEEVAEEVAEEVVEEGEKVPEEVAEEVAEEVAEELVEKVDEQVAEELVEKVDEQVAEELVEKVDEQVVEEVADELVEKVDEEVVEKVDEEVVEEVADELVEKVVEEEGEKVPEEVVEEVAEEV
AEEVVEEGEKVPEEVAEEVAEEVAEEVAEELVEKVDEEVAEKVVEEEGEKVLEEVIEEVVEEVAEEVAEKVVEEQGEK

C-terminal

VNKNDLNDASSEEIKDSSDFKESHEELFKVFLELINKNDLVKENLKKITNNLNEMHLSTLYP

P. falciparum, SD01 isolate (546)

N-terminal

MLNIFNIIFLLFLINIYICEANGTLSENIESAEEIDALKTNLRNGYLNNTYFNEENNNLNIENEINNTNYNEVTEETKEELYDINENIFPDYFFLDIFTENKEQKNEEVPMKIEVVNDGEEVKTEYVSEKNEEVENKSETEIG

Central Repeat

EELTEKVDEKVPEEVAEELVEKVDEEVAEELVEKVDEKVAEEVDQKVDEEVTEELIEKVDEEVTEELIEKVDEEVAEELIEKVDEEVAEELIEKVADELVEKVAEELVEKVDEQVAEELVEKVDEQVAEELVEKVDEQVVEEV
ADELVEKVDEEVVEKVPEEVVEEVAEEVAEEVVEEGEKVPEEVAEEVAEEVAEEVAEELVEKVDEQVAEELVEKVDEQVAEELVEKVDEQVVEEVADELVEKVDEEVVEKVDEEVVEEVADELVEKVVEEEGEKVPEEVVEEV
AEEVAEELVEKVDEEVAEKVVEEEGEKVLEEVIEEVVEEVAEEVAEKVVEEQGEK

C-terminal

VNKNDLNDASSEEIKDSSDFKESHEELFKVFLELINKNDLVKENLKKITNNLNEMHLSTLYP

Supplementary Figure S3A. continued

P. falciparum, SN01 isolate (668)

N-terminal

MLNIFNIIFLLFLINIYICEANGTLSENIESAEEIDALKTNLRNGYLNNTYFNEENNNLNIENEINNTNYNEVTEETKEELYDINENIFPDYFFLDIFTENKEQKNEEVPMKIEVVNDGEEVKTEYVSEKNEEVENKSETEIG

Central Repeat

EELTEKVDEKVPEEVAEELVEKVDEEVAEELVEKVDEKVAEEVDQKVDEEVTEELIEKVDEEVTEELIEKVDEEVAEELIEKVDEEVAEELIEKVADELVEKVAEELVEKVDEQVAEELVEKVDEQVAEELVEKVVEEEGEKV
PEEVVEEVAEEVAEEVVEEGEKVPEEVAEEVAEEVAEELVEKVDEQVAEELVEKVDEQVAEELVEKVDEQVVEEVADELVEKVDEEVVEKVDEEVVEEVADELVEKVVEEEGEKVPEEVVEEVAEEVAEEVVEEGEKVPEEVA
EEVAEEVAEELVEKVDEQVAEELVEKVDEQVAEELVEKVDEQVVEEVADELVEKVDEEVVEKVDEEVVEEVADELVEKVVEEEGEKVPEEVVEEVAEEVAEEVVEEGEKVPEEVAEEVAEEVAEEVAEELVEKVDEEVAEKVV
EEEGEKVLEEVIEEVVEEVAEEVAEKVVEEQGEK

C-terminal

VNKNDLNDASSEEIKDSSDFKESHEELFKVFLELINKNDLVKENLKKITNNLNEMHLSTLYP

P. falciparum, TG01 isolate (593)

N-terminal

MLNIFNIIFLLFLINIYICEANGTLSENIESAEEIDALKTNLRNGYLNNTYFNEENNNLNIENEINNTNYNEVTEETKEELYDINENIFPDYFFLDIFTENKEQKNEEVPMKIEVVNDGEEVKTEYVSEKNEEVENKSETEIG

Central Repeat

EELTEKVDEKVPEEVAEELVEKVDEEVAEELVEKVDEKVAEEVDQKVDEEVTEELIEKVDEEVTEELIEKVDEEVAEELIEKVDEEVAEELIEKVADELVEKVAEELVEKVDEQVAEELVEKVDEQVAEELVEKVDEQVDEEL
VEKVDEQVVEEVADELVEKVDEEVVEKVDEEVVEEVADELVEKVVEEEGEKVPEEVVEEVAEEVAEEVVEEGEKVPEEVPEEVAEEVAEEVAEELVEKVDEEVVEKVDEEVVEEVADELVEKVVEEEGEKVPEEVVEEVAEEV
AEEVVEEGEEVAEEVAEEVAEEVAEELVEKVDEEVAEKVVEEEGEKVLEEVIEEVVEKVDEEVAEKVVEEEGEKVLEEVIEEVVEEVAEEVAEKVVEEQGEK

C-terminal

VNKNDLNDASSEEIKDSSDFKESHEELFKVFLELINKNDLVKENLKKITNNLNEMHLSTLYP

Supplementary Figure S3B. N- and C-terminal semi-conserved and armadillo repeat sequences for *Pf*MAAP in five Laverania subgenus of Plasmodium (*P.rechenowi*, PRG01_1034400 and PRCDC_1035200; *P.billcolllinsi*, PBILCG01_1034800; *P. gaboni*, PGSY75_0012400; *P.praefalciparum*, PPRFG01_1036900; and *P. adleri*, PADL01_1034600. All sequences were obtained from Plasmodb (https://plasmodb.org/plasmo/).

>PRG01_1034400 | Plasmodium reichenowi G01 | probable protein, unknown function | protein | length=571

N-terminal

MLNIFNIIFLLFLINIYICEANGALSENIESAEEIDTLKTNLRNGYLNNTYFNEENNNLNIGNEINNTNYNEVTEETKEELYDINQNIFPDYFFLDIVPEKKEQKNEEVPMKIEVVNDGEEVKTESATEKNEEVENKSATEIG

Central Repeat

EEVVEKVDEKVDEKVDEKVDEEVVEELVEEEGEEVAEELIEKVDEEVADEVAEEVAEEVAEEIDEEVVEELIEKVDEKVPEEVVEEVVEEVADEVADGVAEEIDEEVVEEVVEEVADEVAEEVVEEVADEVAEELIEKVDEEV
ADEVAEEVAEEVAEEIDEEVVEELIEKVDEKVPEEVVEEVVEEVADEVADGVAEEIDEEVVEELIEKVDEKVPEEVVEEVVEEVDEEVDEKVPEEVVEEVADEVADGVAEEVVEEVVEELIEQVDEKVAEEVVEEVVEEVAEK
LVEEEGEKVPEEVAEKVVQEEGEKVPEEVVEEVAEEVAEEVAEEVAEEVAEKVVEEVAEEVAEKVVEEVAEKVVEE

C-terminal

EGEKVNKNDLNDAASEEIKDSSDFKESHEELFKVFMELINKNDLVKENLKKLTNTLNEMNISTLYP

>PRCDC_1035200 | Plasmodium reichenowi CDC | probable protein, unknown function | protein | length=545

N-terminal

MLNIFNIIFLLFLINIYICEANGALSENIESAEEIDTLKTNLRNGYLNNTYFNEENNNLNIGNEINNTNYNEVTEETKEELYDINQNIFPDYFFLDIVPENKEQKNEEVPMKIEVVNDGEEVKTESATEKNEEVENKSATEIG

Central Repeat

EEVDEKVDEKVDEKVDEKVDGEVVEEVAEEVADEVAEEVADEVAEEVVQTVDEEVVEELIEKVDEKVDEEVADEIDEEVAEEMDEEVVEELIEKVDESVAEVVEEVVEEVAEEVVEEEGEKLVEEEGEEVAEELIEKVDEEVA
EEVAEEVAEEVVQTVDEEVVEELIEKVDEKVDEEVADEVADGVAEEIDEEVVEELIEKVDEKVPEEVVEEVVEEVDEKVPEEVVEVVEEVADEVADGVAEEVVEEVVEELIEQVDEKVAEEVVEEVVEEVAEKLVEEEGEKVP
EEVAEKVVQEEGEKVPEEVVEEVAEEVAEEVAEEVAEKVVEEVAEKVIEE

C-terminal

EGEKVNKNDLNDAASEEIKDSSDFKESHEELFKVFMELINKNDLVKENLKKLTNTLNEMNISTLYP

Supplementary Figure S3B. continued

>PBILCG01_1034800 | Plasmodium billcollinsi G01 | probable protein, unknown function | protein | length=394

N-terminal

MMNIFYIIFLLFLINIYTCETIGALSENVESVEEIDTLKTNLRNGNLNNTYFNEENNNLNIGNEINNTNYNEVTEETKEELYDINENIFPEYFFLDIVTENKEQKNEEVSMKTEVVNDGEEVGDESIIEEEVETESITEENEE
VETESVTEEN

Central Repeat

EEVETESVIEIAEKVPEEVVEEVAEEVPEVVEEVVEEVAEEVPEVVEEVVEEVAEEVVEEVVEEVVEEVVEEVPEKMPEEVPEKVSEEVPEKVSEEVPEKVSEEVPEEVHEEAPEEVVEEVAEKIPEAVEDAAEKIPEAVEDVAE
KIPEVVEDVAEEVVEEVAEKIPEVVEDVAEKV

C-terminal

EKINKNDLNDAASEEIKDSSDFKESHEELFKVFLELIDKNDLVKENLKKLTKILNEINLSTIYP

PGSY75_0012400 | Plasmodium gaboni strain SY75 | putative protein, unknown function | protein | length=207

N-terminal

MMNFFYIIFLLFLINLYICETYGALSENIESAEEIDTLKTSLRNGHLNNTYFNEENNNLNIGNEINNTNYKEVTEESKEELYDINENIFPDYFFLDIVTENQEQKNEEVPVKTEVVSDE

Central Repeat

EELETESVIEEVEVETESINDEGETESVIEEIEIETESINDEGETETVIEEIEIETESINDEGETETVIEEIEIETESIN

C-terminal

SSDLKESH

Truncated?

PPRFG01_1036900 | Plasmodium praefalciparum strain G01 | probable protein, unknown function, putative | protein | length=602

N-terminal

MLNIFNIIFLLFLINIYICEANGTLSENIESAEEIDALKTNLRNGYLNNTYFNEENNNLNIENEINNTNYNEVTEETKEELYDINENIFPDYFFLDIFTENKEQKNEEVPMKIEVVNDGEEVKTEYVSEKNEEVENKSETEIG

Central Repeat

EELTEKVDEIVDEKVPEEVAEVVEEVPEEVDEVTEELIEKVDEEVTEELIEKVDEKVTEELIEKVDEKVPEEVADEVVEEVVEEVADELVEKVVEEVVEEVDEVAEVAEEVDQKVDEEVTEELIEKVDEKVDEEVAEELIEKV
AEELVEKVDEKVADELVEKVDEEVAEEVVEEVAEEVAEELIEKVDEEVVEKVDEEVAEELVEELIEKVDEEVAEELIEKVDEEVVEEVADELVEKVDEEVVEEVADELVEKVDEEVVEEVADELVEKVDEEVAEEVVEEEGEK
VAEEVVEEVAEEVAEEVAEEVVEEGEKVPEEVVEEVAEELVEKVDEEVAEEVAEEVAEEVAEELVEKVDEEVVEEVAEKVVEEEGEKVLEEVIEEVVEEVAEEVAEKVVE

C-terminal

EEGEKVNKNDLNDACFRASDFKESHEELFKVFLELINKNDLVKENLKKLTNNLNEMHLSTLYP

Supplementary Figure S3B. continued

>PADL01_1034600 | Plasmodium adleri G01 | probable protein, unknown function | protein | length=354

N-terminal

MMNFFYIIFLLFLINLYICETNGALSENIESAEEIDTLKTSLRNGYLNNTYFNEEKNNLNIGNEINNTNYNEVTQESKEELFDINENISPDYFFLDIVTENKEQKNEEVPVKTEVVSNE

Central Repeat

EELETESVIEEVEVETESISDEEVETESISDEEVETESVTEEVEVETESINDEGETESVIEEVEVETESINDEGETESVIEEIEIETESINDEEGETESVIEEIEIETESINDEGETESVIEEIEIETESINDEEGETESVIE
EIEIETESINDEEVETESVIEEIEIETESINDEEEVETESVTEEVEVETESINDEEGETESVAAVV

C-terminal

EKVNKNDLNDAASEEIKDSSDFKESH

truncated sequences
