# Supplementary figures and images for: Plasmodium falciparum Merozoite Associated Armadillo Protein (PfMAAP) Is Apically Localized in Free Merozoites and Antibodies Are Associated With Reduced Risk of Malaria
Source: Front Immunol. 2020 Apr 7;11:505. doi: 10.3389/fimmu.2020.00505 (PMC7155890; doi:10.3389/fimmu.2020.00505)

Figure S4

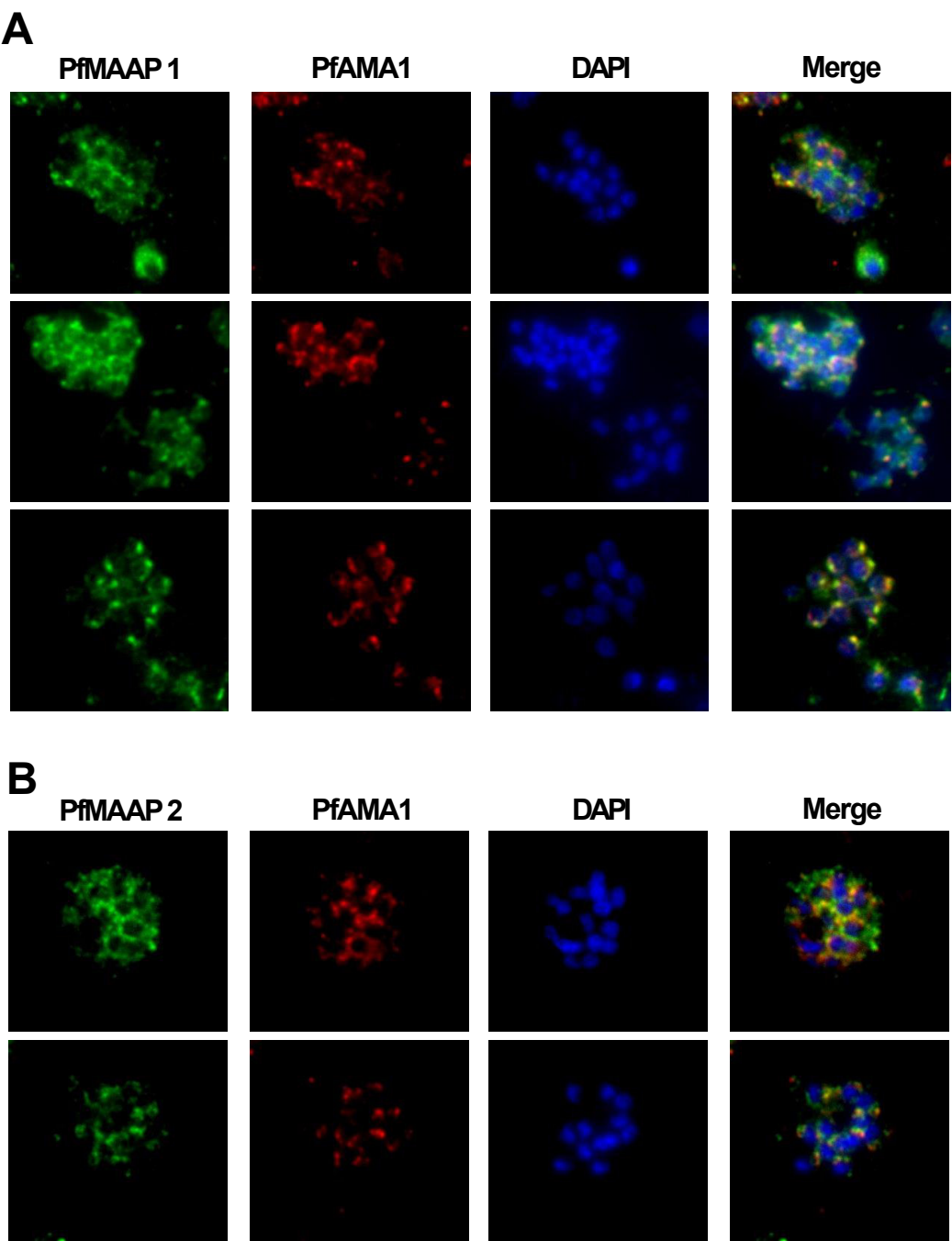

Supplement: Figure S4 — Co-localization of α-PfMAAP1 and 2 with α-AMA1. (A) PfMAAP1 (green) localization pattern relative to PfAMA1 (red) with DAPI (blue) staining for nuclei and the merging of all channels (Merge). (B) PfMAAP2 (green) co-localization with PfAMA1 (red) with DAPI (blue) staining the nuclei and the merge of all channels (Merge). Fifty images were taken per antibody tested using an Olympus model BX41 fluorescent microscope with a x100 oil-immersion objective. [file Image_4.pdf]
