# Supplementary material for: Plasmodium falciparum Merozoite Associated Armadillo Protein (PfMAAP) Is Apically Localized in Free Merozoites and Antibodies Are Associated With Reduced Risk of Malaria
Source: Front Immunol. 2020 Apr 7;11:505. doi: 10.3389/fimmu.2020.00505 (PMC7155890; doi:10.3389/fimmu.2020.00505)
Supplement: Supplementary file 5 [file Table_1.pdf]

Table S1. Deduced amino acid sequence information for the 16 *Plasmodium falciparum* isolates surveyed in the study.

Plasmodb ID	Total size of protein	Size of repeat region	Position of repeats	Country of origin
PF3D7_1035900	566	360	144-504	lab isolate
PfSD01_100040400	546	340	144-484	Sudan
PfML01_100039900	569	363	144-507	Mali
PfKE01_100041100	565	359	144-503	Kenya
PfIT_100039800	447	241	144-385	lab isolate
PfGB4_100040700	672	466	144-610	lab isolate
PfGN01_100041300	569	363	144-507	Guinea
PfTG01_100041000	593	387	144-531	Togo
PfDd2_100041100	557	351	144-495	lab isolate
PfSN01_100041200	668	462	144-606	Senegal
PfKH02_100041200	557	351	144-495	Cambodia
Pf7G8_100040200	371	165	144-309	lab isolate
PfHB3_100040200	565	359	144-503	lab isolate
PfGA01_100041100	565	359	144-503	Gabon
PfKH01_100040300	569	363	144-507	Cambodia
PfCD01_100041000	569	363	144-507	Congo

Note: Isolates were reported in Otto et al. 2018 (PMID: 29784978).
