# Supplementary material for: Plasmodium falciparum Merozoite Associated Armadillo Protein (PfMAAP) Is Apically Localized in Free Merozoites and Antibodies Are Associated With Reduced Risk of Malaria
Source: Front Immunol. 2020 Apr 7;11:505. doi: 10.3389/fimmu.2020.00505 (PMC7155890; doi:10.3389/fimmu.2020.00505)
Supplement: Supplementary file 6 [file Table_2.pdf]

Table S2. Deduced amino acid sequence information for PfMAAP for the *Laverania* species compared to the 3D7 reference isolate.

ID	Size of protein	Size of repeat	Repeat coordinates	Isolate	**References**
PF3D7_1035900	566	362	144-506	Laboratory isolate	1
PRG01_1034400	571	367	144-511	*P. reichenowi* G01 isolate	2
PRCDC_1035200	545	341	144-485	*P. reichenowi* CDC isolate	3
PBILCG01_1034800	394	192	142-334	*P. billcollinsi* G01 isolate	2
PGSY75_0012400*	207	n/a	n/a	*P.gaboni* SY75 isolate	3
PPRFG01_1036900	602	402	144-546	*P. praefalciparum* G01 isolate	4
PADL01_1034600	354	190	142-332	*P. adleri* G01 isolate	5

Note: All isolates had a repeat periodicity of 3.92 with the exception of *P.adleri* and *P.gaboni*. The asterisk indicates that the database sequence for *P.gaboni* was deposited as a truncated sequence.

References:

1. Otto TD, Böhme U, Sanders M, Reid A, Bruske EI, Duffy CW, Bull PC, Pearson RD, Abdi A, Dimonte S, Stewart LB, Campino S, Kekre M, Hamilton WL, Claessens A, Volkman SK, Ndiaye D, Amambua-Ngwa A, Diakite M, Fairhurst RM, Conway DJ, Franck M, Newbold CI, Berriman M. Long read assemblies of geographically dispersed Plasmodium falciparum isolates reveal highly structured subtelomeres. Wellcome Open Res. 2018 May 3;3:52. doi: 10.12688/wellcomeopenres.14571.1.
2. Otto TD, Gilabert A, Crellen T, Böhme U, Arnathau C, Sanders M, Oyola SO, Okouga AP, Boundenga L, Willaume E, Ngoubangoye B, Moukodoum ND, Paupy C, Durand P, Rougeron V, Ollomo B, Renaud F, Newbold C, Berriman M, Prugnolle F. Genomes of all known members of a Plasmodium subgenus reveal paths to virulent human malaria. Nat Microbiol. 2018 Jun;3(6):687-697. doi: 10.1038/s41564-018-0162-2.
3. Sundararaman SA, Plenderleith LJ, Liu W, Loy DE, Learn GH, Li Y, Shaw KS, Ayouba A, Peeters M4, Speede S, Shaw GM, Bushman FD, Brisson D, Rayner JC, Sharp PM, Hahn BH. Genomes of cryptic chimpanzee Plasmodium species reveal key evolutionary events leading to human malaria. Nat Commun. 2016 Mar 22;7:11078. doi: 10.1038/ncomms11078.
4. Gilabert A, Otto TD, Rutledge GG, Franzon B, Ollomo B, Arnathau C, Durand P, Moukodoum ND, Okouga AP, Ngoubangoye B, Makanga B, Boundenga L, Paupy C, Renaud F, Prugnolle F, Rougeron V. Plasmodium vivax-like genome sequences shed new insights into Plasmodium vivax biology and evolution. PLoS Biol. 2018 Aug 24;16(8):e2006035. doi: 10.1371/journal.pbio.2006035.
5. Liu W, Sundararaman SA, Loy DE, Learn GH, Li Y, Plenderleith LJ, Ndjango JB, Speede S, Atencia R, Cox D, Shaw GM, Ayouba A, Peeters M, Rayner JC, Hahn BH, Sharp PM. Multigenomic Delineation of Plasmodium Species of the Laverania Subgenus Infecting Wild-Living Chimpanzees and Gorillas. Genome Biol Evol. 2016 Jul 2;8(6):1929-39. doi: 10.1093/gbe/evw128.
