# Supplementary material for: Plasmodium falciparum Merozoite Associated Armadillo Protein (PfMAAP) Is Apically Localized in Free Merozoites and Antibodies Are Associated With Reduced Risk of Malaria
Source: Front Immunol. 2020 Apr 7;11:505. doi: 10.3389/fimmu.2020.00505 (PMC7155890; doi:10.3389/fimmu.2020.00505)
Supplement: Supplementary file 7 [file Table_3.pdf]

Table S3: Elisa results of pre-immune serum and purified antibody

	Dilutions from 1mg/ml	Antibody Conc (ng/ml)	PfMAAP1	PfMAAP2	PfMAAP 3
NC	1:1000	NA	0.073	0.075	0.101
1	1:1000	1000.00	3.749	3.347	3.229
2	1:2000	500.00	3.561	3.270	3.019
3	1:4000	250.00	3.397	3.164	2.968
4	1:8000	125.00	3.186	2.882	2.656
5	1:16000	62.50	2.938	2.649	2.433
6	1:32000	31.25	2.552	2.192	1.907
7	1:64000	15.62	2.050	1.671	1.206
8	1:128000	7.81	1.312	1.018	0.625
9	1:256000	3.90	0.695	0.574	0.324
10	1:512000	1.95	0.364	0.289	0.175
11	Blank	Blank	0.049	0.043	0.056
12	Blank	Blank	0.049	0.043	0.056

The titer is the highest dilution with S/B(Signal/Blank) >=2.1

NC is negative control (Pre-immune serum)
